# Stimulus Selection Influences Prediction of Individual Phenotypes in Naturalistic Conditions

**DOI:** 10.1002/hbm.70164

**Published:** 2025-02-17

**Authors:** Xuan Li, Simon B. Eickhoff, Susanne Weis

**Affiliations:** ^1^ Institute of Neuroscience and Medicine, Brain and Behaviour (INM‐7), Research Centre Jülich Jülich Germany; ^2^ Institute of Systems Neuroscience Medical Faculty, Heinrich Heine University Düsseldorf Düsseldorf Germany

**Keywords:** behaviour prediction, individual differences, movie stimuli, naturalistic viewing, sex classification, stimulus selection

## Abstract

While the use of naturalistic stimuli such as movie clips for understanding individual differences and brain–behaviour relationships attracts increasing interest, the influence of stimulus selection remains largely unclear. By using machine learning to predict individual traits (phenotypes) from brain activity evoked during various movie clips, we show that different movie stimuli can result in distinct prediction performances. In brain regions related to lower‐level processing of the stimulus, prediction to a certain degree benefits from stronger synchronisation of brain activity across subjects. By contrast, better predictions in frontoparietal brain regions are mainly associated with larger inter‐subject variability. Furthermore, we demonstrate that while movie clips with rich social content in general achieve better predictions, the importance of specific movie features for prediction highly depends on the phenotype under investigation. Overall, our findings underscore the importance of careful stimulus selection and provide novel insights into stimulus selection for phenotype prediction in naturalistic conditions, opening new avenues for future research.

## Graphical abstract

Stimulus selection is crucial for phenotype prediction in naturalistic conditions. The level of inter‐subject synchrony in brain activity strongly influences the predictive capability, with the effect largely following the functional hierarchy of the brain. Relevant features of movie stimuli for prediction highly depend on the phenotype under investigation.

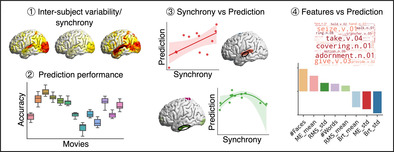

## 1 Introduction

Understanding inter‐individual variability in brain function in relation to behaviour is a central goal of human neuroscience. Naturalistic stimuli, for example, movies or spoken stories, have recently emerged as a promising tool for advancing this goal. They introduce more synchrony in brain activity across subjects relative to unconstrained resting‐state conditions (Hasson et al. 2004), mitigating unsystematic noises and improving the interpretability of measured brain signals (Vanderwal et al. 2015). They also provide more ecological validity than conventional tasks by engaging the brain in conditions more similar to real‐world situations (Sonkusare, Breakspear, and Guo 2019), offering novel insights into complex cognitive processes, such as the hierarchical organisation of memory (Hasson, Chen, and Honey 2015) and naturalistic emotions (Nummenmaa et al. 2012). While most studies have used naturalistic stimuli for investigating brain activity patterns shared across subjects, an increasing number of studies have suggested their utility for studying individual differences. For example, previous studies have found that naturalistic stimuli evoke reliable individual differences in brain activity, promoting individual identification and showing associations with stable personal traits and mental disorders (Byrge et al. 2015; Campbell et al. 2015; Finn et al. 2020; Vanderwal et al. 2017).

Simultaneously, studies of individual differences and brain–behaviour relationships have been greatly facilitated by the application of machine learning (ML) algorithms. Unlike traditional correlation analysis, ML allows for making predictions at an individual subject level on novel samples from nuanced patterns in brain data (Dubois and Adolphs 2016; Gabrieli, Ghosh, and Whitfield‐Gabrieli 2015; Yarkoni and Westfall 2017). By adopting such ML‐inspired predictive frameworks, many studies have revealed the associations between brain activity and a variety of personal traits (Dubois et al. 2018; Hsu et al. 2018), behaviours (Avery et al. 2020), and clinical symptoms (Drysdale et al. 2017). This has facilitated our understanding of neural correlates of human cognition and behaviour, and may also advance biomarker discovery for mental disorders and personalised treatments in clinical applications (Rosenberg, Casey, and Holmes 2018; Woo et al. 2017).

Understanding individual differences in naturalistic conditions with ML‐based predictive frameworks presents a natural and promising choice for future studies. Recent studies have shown that naturalistic viewing conditions outperform resting or conventional task conditions in predicting individual phenotypes (Finn and Bandettini 2021; Li et al. 2023). Additionally, naturalistic conditions may help mitigate the need for large sample sizes for prediction (Rosenberg and Finn 2022; Schulz et al. 2024), complementing other methodological approaches (He et al. 2022; He et al. 2024). However, different naturalistic stimuli may not work equally well. The elicited patterns of brain activity might seem broadly similar across stimuli at first glance, with strong responses in visual and auditory sensory cortices (Hasson et al. 2004), but they are nevertheless not identical. For example, it has been shown that the brain responds differently to stimuli that differ in the expressed emotions or the story timeline (Gruskin, Rosenberg, and Holmes 2020; Hasson, Yang et al. 2008; Kauttonen et al. 2018). Individuals' brain functional networks have also been shown to vary across different stimuli (Kröll et al. 2023; Vanderwal et al. 2017). Convergingly, it has become clear that stimulus selection is of great importance in naturalistic studies. Selecting an appropriate stimulus allows us to better use the potential of naturalistic conditions for studying brain–behaviour relationships, especially in clinical settings where patient groups may be highly sensitive to the choice of stimulus (Eickhoff, Milham, and Vanderwal 2020). However, it remains largely unclear how the choice of stimulus influences the utility of naturalistic conditions for phenotype prediction.

**FIGURE 1:**
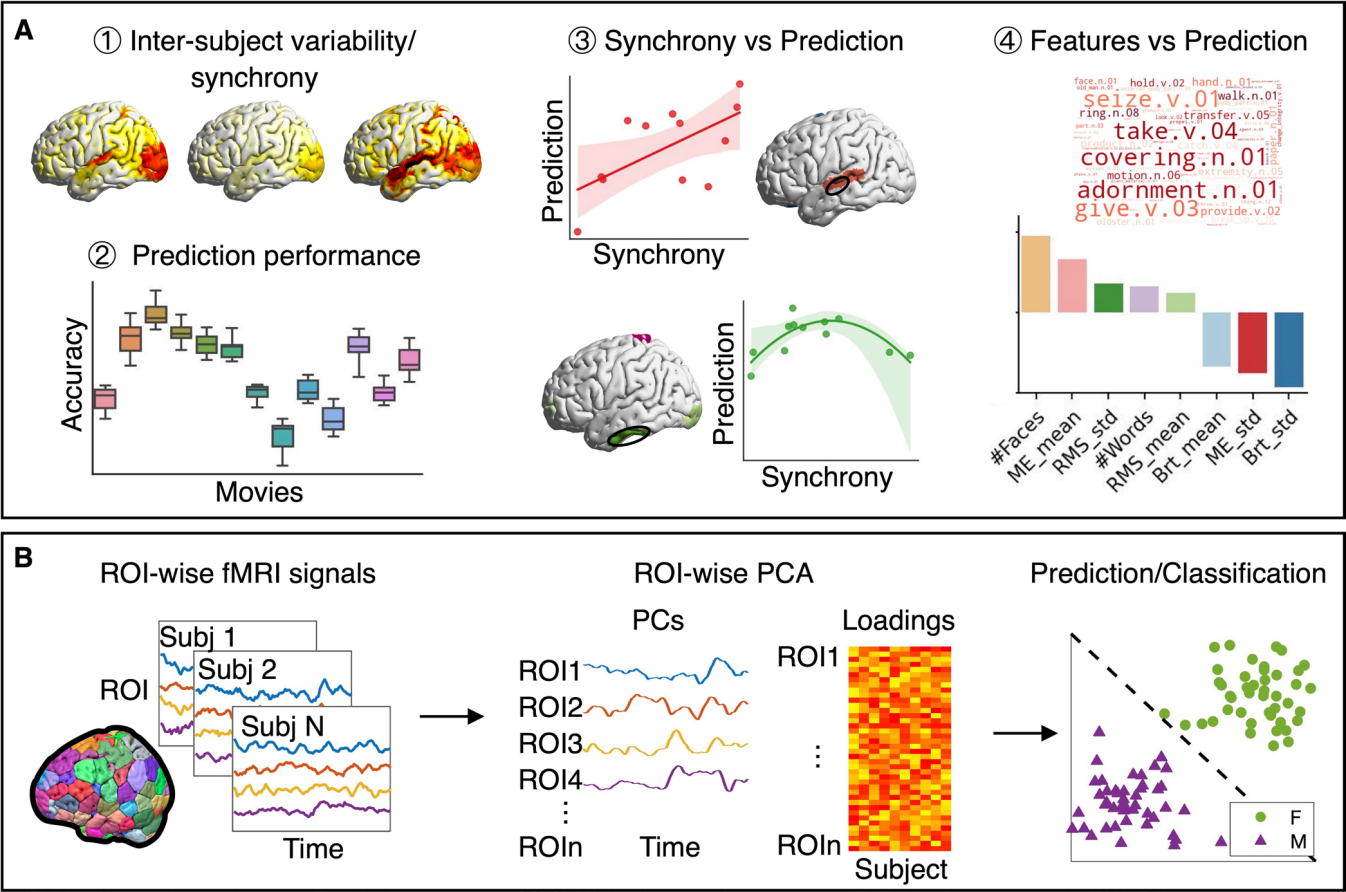
Analysis framework for understanding influence of stimulus selection on phenotype prediction with TOPF. (A) Analysis pipeline for investigating the influence of stimulus selection on phenotype prediction in naturalistic conditions. We examined ① differences in inter‐subject variability/synchrony in evoked brain activity across movie clips, ② differences in phenotype prediction performance and predictive models across movie clips, ③ the relationship between inter‐subject variability/synchrony in evoked brain activity and prediction performance, and ④ the influence of movie features on prediction performance. (B) Schematic illustration of the TOPF method. Image adapted from (Li et al. 2023). Given a brain parcellation, the mean fMRI time series over voxels is extracted for each region of interest (ROI). A principal component analysis (PCA) is applied to the fMRI time series across subjects for each ROI separately. The derived PCs represent shared responses across subjects, with PC loadings reflecting individual expression levels of the shared responses. The PC loadings are later used as features for ML‐based phenotype prediction (see Section 15 for detailed descriptions of the method).

In the present study, we aim to systematically evaluate the influence of different movie stimuli on phenotype prediction. We examined (i) inter‐subject variability/synchrony in brain activity, (ii) prediction performance and predictive models of individual phenotypes during different movie clips, (iii) the influence of inter‐subject synchrony on prediction performance, and (iv) the influence of various features of movie stimuli on prediction performance (Figure 1A). We hypothesise that the capability for phenotype prediction depends on the choice of the movie stimulus. Additionally, we hypothesise that stimulus selection for phenotype prediction could be facilitated by a better understanding of the influence of inter‐subject variability/synchrony in brain activity and features of the movie stimuli.

For phenotype prediction, we used our recently proposed approach, the topography‐based predictive framework (TOPF) (Li et al. 2023). Brain signals recorded during naturalistic conditions are highly complex, containing stimulus‐evoked brain responses, spontaneous brain activity, and noise. The inter‐subject correlation (ISC) approach is a fundamental tool in naturalistic studies for examining stimulus‐evoked responses (Hasson et al. 2004; Nastase et al. 2019; Simony et al. 2016). The core idea of this family of approaches is that any consistent, synchronous fluctuations in brain activity across subjects (i.e., inter‐subject synchrony) result from processing the same stimulus. ISC approaches have been shown to be useful not only for understanding brain responses shared across subjects but also for studying individual differences (Chen et al. 2020; Finn et al. 2020). The TOPF approach builds upon similar core concepts of ISC with a special focus on individual differences, which are represented by individual expression levels of shared responses (Figure 1B). Furthermore, TOPF allows us to investigate the brain–behaviour relationship in a ML‐based predictive framework, which has been shown to be useful for naturalistic studies (Li et al. 2023).

Here, we used functional magnetic resonance imaging (fMRI) data acquired while subjects were watching 13 different movie clips from the Human Connectome Project (HCP) dataset (Van Essen et al. 2013). First, we quantified inter‐subject variability/synchrony in brain activity for each brain region. We examined whether and how individual differences in response to the same stimulus vary across conditions and brain regions. Second, we used brain activity evoked during different movie clips to predict two well‐studied phenotypes in fMRI studies, biological (i.e., birth‐assigned) sex and fluid intelligence, with TOPF across different ML settings. We investigated whether and how prediction performance and predictive models vary across movie clips. Third, we explicitly modelled the relationship between prediction performance and inter‐subject synchrony of brain activity across movie clips for each brain region. This addressed an important question in naturalistic studies: how does the level of synchrony elicited while watching a movie clip influence its utility for phenotype prediction? Finally, we delved into a variety of low‐ and high‐level features of movie stimuli and explored their influence on prediction performance.

## 2 Results

In this study, we used naturalistic viewing fMRI data (*n* = 178) in the HCP 7T subset, where 13 short movie clips (2–5 min in length) covering different topics were presented during the scanning (Table S1). For our analysis, all clips were trimmed to the same length (132 TRs, i.e., 2:12 min). Each clip originated from either Hollywood films (“inception,” “social net,” “ocean 11,” “home alone,” “brockovich,” and “star wars”) or independent films (“two men,” “bridgeville,” “pockets,” “flower,” “hotel,” “garden,” and “dreary”). We conducted four main analyses, detailed in the results section. First, we characterised inter‐subject synchrony of brain activity to investigate variations in inter‐subject variability in elicited brain responses across movie clips. Second, we used the TOPF approach to examine differences across movie clips in their ability to predict individual phenotypes. Third, we analysed the relationship between inter‐subject synchrony and prediction performance across movie clips. Finally, we explored the relationship between various stimulus features and prediction performance across movie clips.

### 2.1 Inter‐Subject Variability/Synchrony in Brain Activity

#### 2.1.1 Inter‐Subject Variability/Synchrony

We measured inter‐subject variability of brain activity evoked during watching movie clips by computing inter‐subject synchrony. Specifically, we extracted mean time series over voxels within each of 400 cortical (Schaefer et al. 2018) and 36 subcortical (Fan et al. 2016) regions of interest (ROIs). For each ROI, we performed a principal component analysis (PCA) to the fMRI time series across subjects. Inter‐subject synchrony was quantified as the variance explained by the first principal component (PC1) (Di and Biswal 2022; Li et al. 2023). A larger value indicates greater synchronisation of brain activity across subjects, thus lower inter‐subject variability.

**FIGURE 2:**
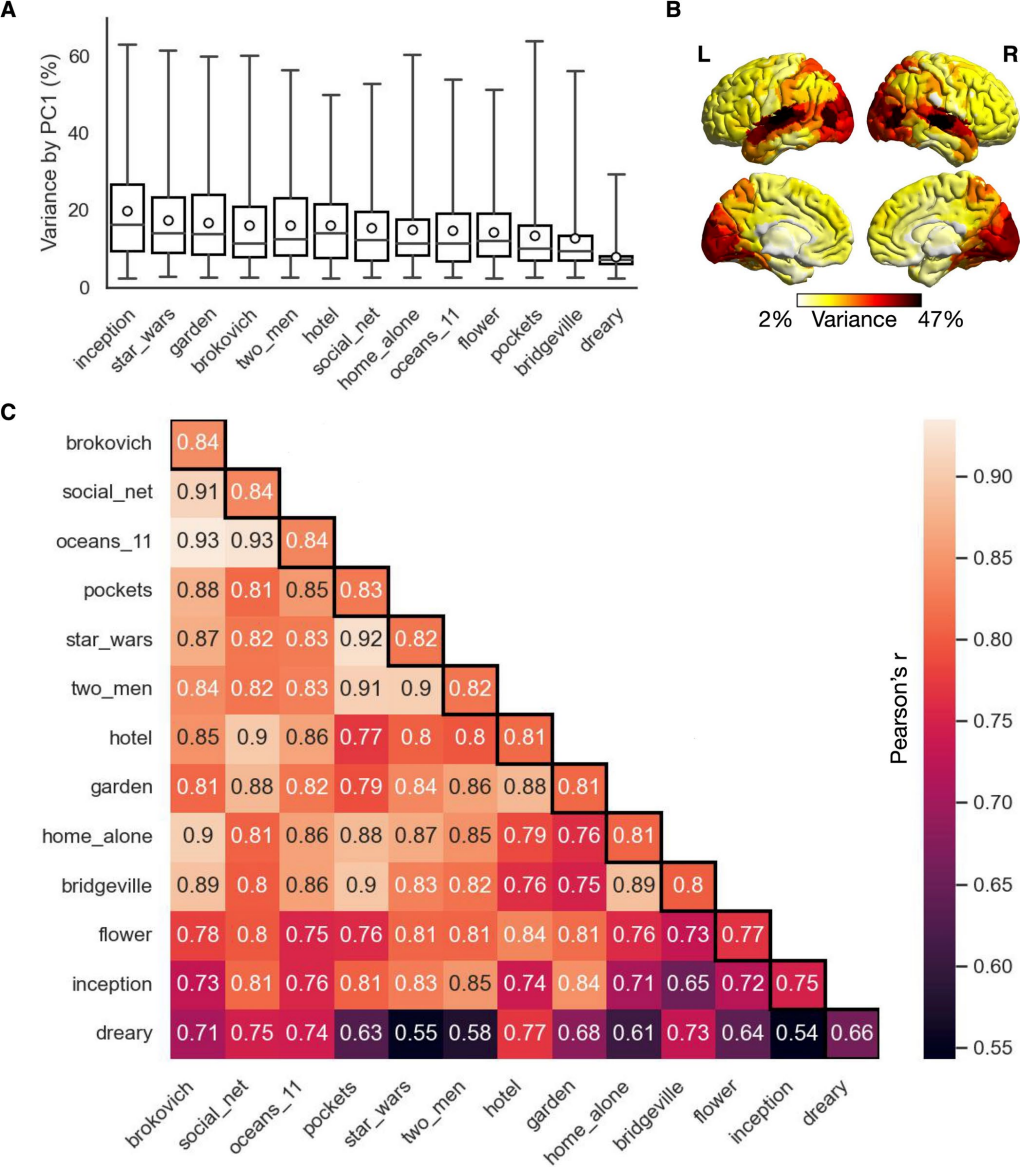
Inter‐subject variability/synchrony of brain activity across different movie clips. (A) Inter‐subject synchrony of brain activity over all ROIs for individual movie clips. The three bars of each box from top to bottom indicate the third quartile, median and first quartile, respectively. The circle in the middle represents the mean. The upper and lower whiskers represent the maximum and minimum, respectively. Movie clips are ordered according to the mean synchrony value over ROIs. (B) Spatial pattern of inter‐subject synchrony across the whole brain. For each ROI, the value represents the mean synchrony value over all movie clips. The light–dark colour bar indicates the synchrony value from low to high. (C) Similarity (Pearson's *r*) between movie clips in spatial patterns of inter‐subject synchrony. Movie clips are ordered according to their average similarity to the other clips (diagonals in black boxes). High and low similarity values are indicated by light and dark colours, respectively.

Figure 2A shows that the inter‐subject synchrony values varied significantly across movie clips (ANOVA: *p* < 0.0001), with the mean value over all ROIs being highest for “inception” (20.9% ± 12.8%) and lowest for “dreary” (7.9% ± 4.0%). The synchrony values also varied largely across the whole brain (max: 63.8%, min: 2.5%), with the temporal and occipital cortices related to audiovisual information processing exhibiting substantially stronger synchrony than the other brain regions (Hasson et al. 2004) (Figure 2B; averaged over all clips). To examine how the spatial pattern of synchrony across the whole brain varied across movie clips, we correlated the synchrony values across ROIs for each pair of movie clips, resulting in a 13 by 13 correlation matrix (Figure 2C). In general, the spatial pattern of synchrony across the whole brain was consistent across movie clips (Pearson's *r* = 0.80 ± 0.05 averaged over all clip pairs; see Figure S1 for synchrony maps of individual movie clips).

Notably, “dreary” was least similar to the other movie clips (average *r* = 0.66 ± 0.08). We further found that brain activity during “dreary” synchronised across subjects only in the visual cortex rather than widely across the sensory cortices, and the synchrony level of “dreary” was much lower than that of the other clips across the whole brain (Figure S1). This might be because “dreary” is the only “non‐social” movie clip in the HCP dataset, containing only natural scenes without human images or speech. In this sense, “dreary” is similar to the well‐known “inscapes” paradigm (Vanderwal et al. 2015), which is also a non‐verbal and non‐social movie clip without narrative. It has been shown that “inscapes” decreases task‐evoked brain activity compared to typical narrative movies and resembles resting state (Kröll et al. 2023; Vanderwal et al. 2017), consistent with our results.

Together, these results show that there was generally larger inter‐subject variability in higher‐order brain regions than in low‐level brain regions related to audiovisual information processing. Moreover, the less‐social movie clip tended to reduce synchrony, thus increasing inter‐subject variability in brain activity.

#### 2.1.2 Variations of Inter‐Subject Variability Across Movie Clips

To further understand how the way subjects differed from each other in brain activity changed across movie clips, we measured the similarity between movie clips of the PC1 loadings over all subjects (i.e., individual expression levels of the shared response) for each ROI. Specifically, a high loading value indicates that brain activity of a given subject closely resembles the shared response. We correlated the PC1 loadings of all subjects between each pair of movie clips within each ROI, resulting in a 13 by 13 correlation matrix for each ROI. A high correlation value indicates that how individual subjects differed in response to a common stimulus is similar between movie clips.

**FIGURE 3:**
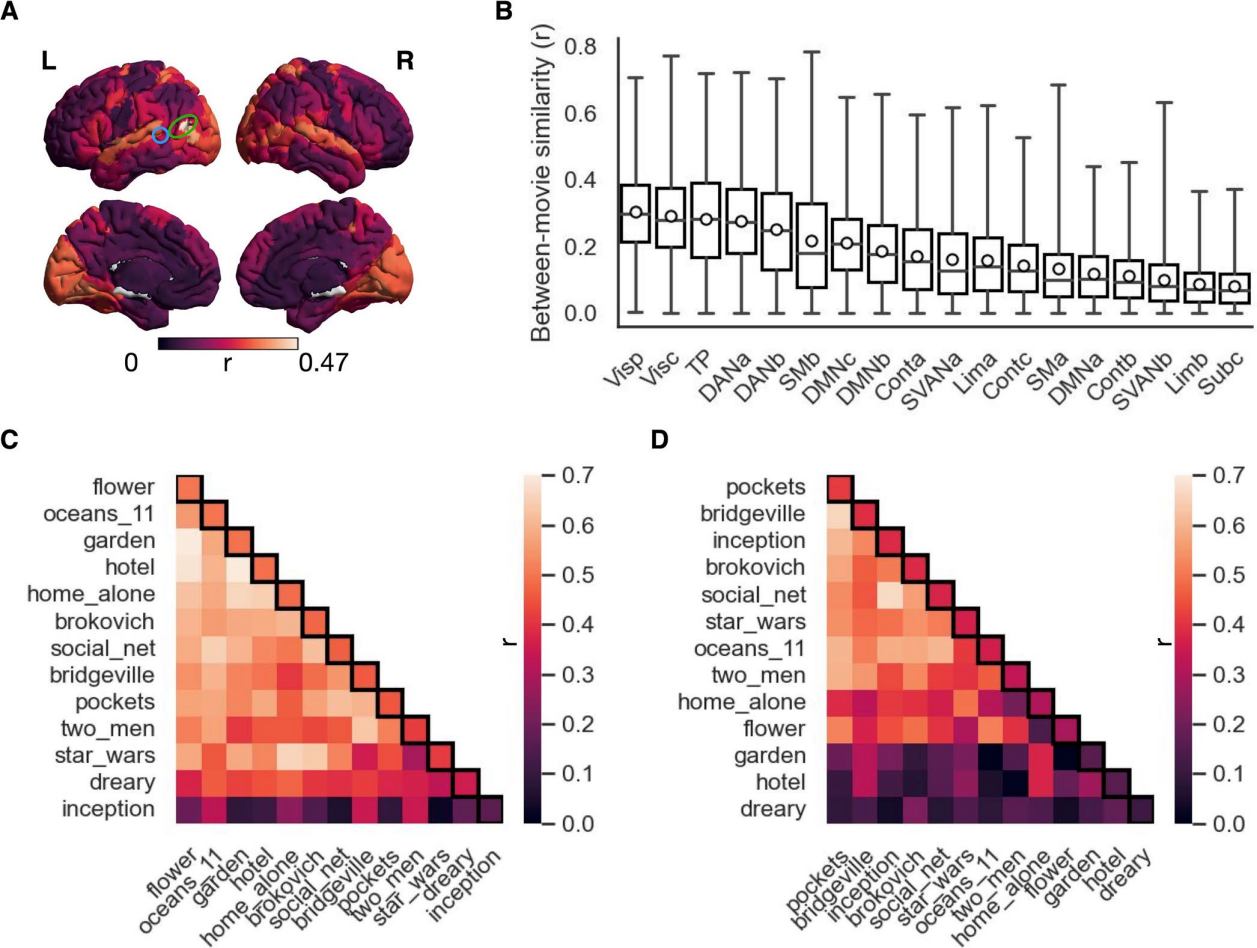
Comparison of individual expression levels of shared responses (PC1 loadings) between movie clips. (A) Between‐movie similarity (Pearson's *r*) of PC1 loadings over all subjects, averaged across all movie clip pairs for each ROI. High and low values are indicated by light and dark colours, respectively. (B) The between‐movie similarity of individual ROIs and movie clip pairs grouped by network. Boxplot convention is consistent with that used in Figure 2A. (C) Correlation matrix for the ROI (marked in a green circle in (A)) achieving the highest average between‐movie similarity. (D) Correlation matrix for the ROI (marked in a blue circle in (A)) achieving the highest standard deviation of the between‐movie similarity. Movie clips are ordered according to their average similarity to the other clips (diagonals in black boxes).

We found that the between‐movie similarity (averaged over all pairs of clips) varied largely across the whole brain (*r* = 0–0.47), with the value being higher in occipital, temporal and parietal cortices than in the other cortices (Figure 3A; see Figure S2 for average maps of individual movie clips). Figure 3B shows the between‐movie similarity for all pairs of movie clips of all ROIs, grouped by network (Yeo et al. 2011). Similarly, we found that the between‐movie similarity was higher in the visual, temporal–parietal and dorsal attention networks than in the frontoparietal network (FPN) and limbic network.

On the other hand, the between‐movie similarity also highly depended on which pairs of movie clips were being compared (*r* = 0–0.79). For example, an ROI in the left temporo‐parieto‐occipital junction (green circle in Figure 3A) achieved the largest average between‐movie similarity (*r* = 0.47 ± 0.16). That is, how subjects differed from each other in brain activity was most consistent across movie clips in this ROI. Here, we found that “inception” and “dreary” achieved the lowest similarity to the other movie clips (Figure 3C; inception: *r* = 0.16 ± 0.11; dreary: *r* = 0.36 ± 0.13). This region has been found to involve multiple functions during naturalistic viewing, such as episodic memory (Hasson, Furman et al. 2008), attention (Langner and Eickhoff 2013), and social perception (Lahnakoski et al. 2012). This finding suggests that the two movie clips evoked distinct patterns of individual differences in these brain processes from the other clips.

As an additional example, an ROI in the superior temporal gyrus (STG) exhibited the most variable patterns of expression levels over subjects across movie clips, with the standard deviation of the between‐movie similarity achieved the highest value (blue circle in Figure 3A; *r* = 0.38 ± 0.20). Here, we found that “garden” (*r* = 0.16 ± 0.11), “hotel” (*r* = 0.16 ± 0.11), and “dreary” (*r* = 0.11 ± 0.06) were least similar to the other movie clips (Figure 3D). STG is known for its crucial role in language and speech processing (Mesgarani et al. 2014). Correspondingly, our further analysis showed that the amount of speech (quantified as the number of spoken words) was lowest for “dreary” and “hotel” and highest for “garden” (Table S2).

Overall, these results show that different movie clips can elicit distinct patterns of individual differences in neural responses both within and beyond cortices for audiovisual information processing.

### 2.2 Prediction of Individual Phenotypes

#### 2.2.1 Prediction Performance

Having shown that different movie clips can elicit distinct patterns of individual differences in brain activity, we investigated whether they also differ in their capability for phenotype prediction. Here we focused on two phenotypes, namely biological sex and fluid intelligence (measured as the number of correct responses of Raven's progressive matrices) (Bilker et al. 2012). We applied the TOPF method (Li et al. 2023) for phenotype prediction for each movie clip separately (Figure 1B). Briefly, we used the PC loadings from all ROIs as features for prediction and evaluated model performance via a 10‐fold cross‐validation (CV). We ensured that training and test subjects in each CV fold were fully separated before feature extraction and that subjects from the same family were all included in either the training or the test set to prevent data leakage. Prediction performance was quantified as balanced accuracy for sex and Pearson's correlation between predicted and observed values for fluid intelligence. We repeated the procedure 10 times to obtain stable results.

**FIGURE 4:**
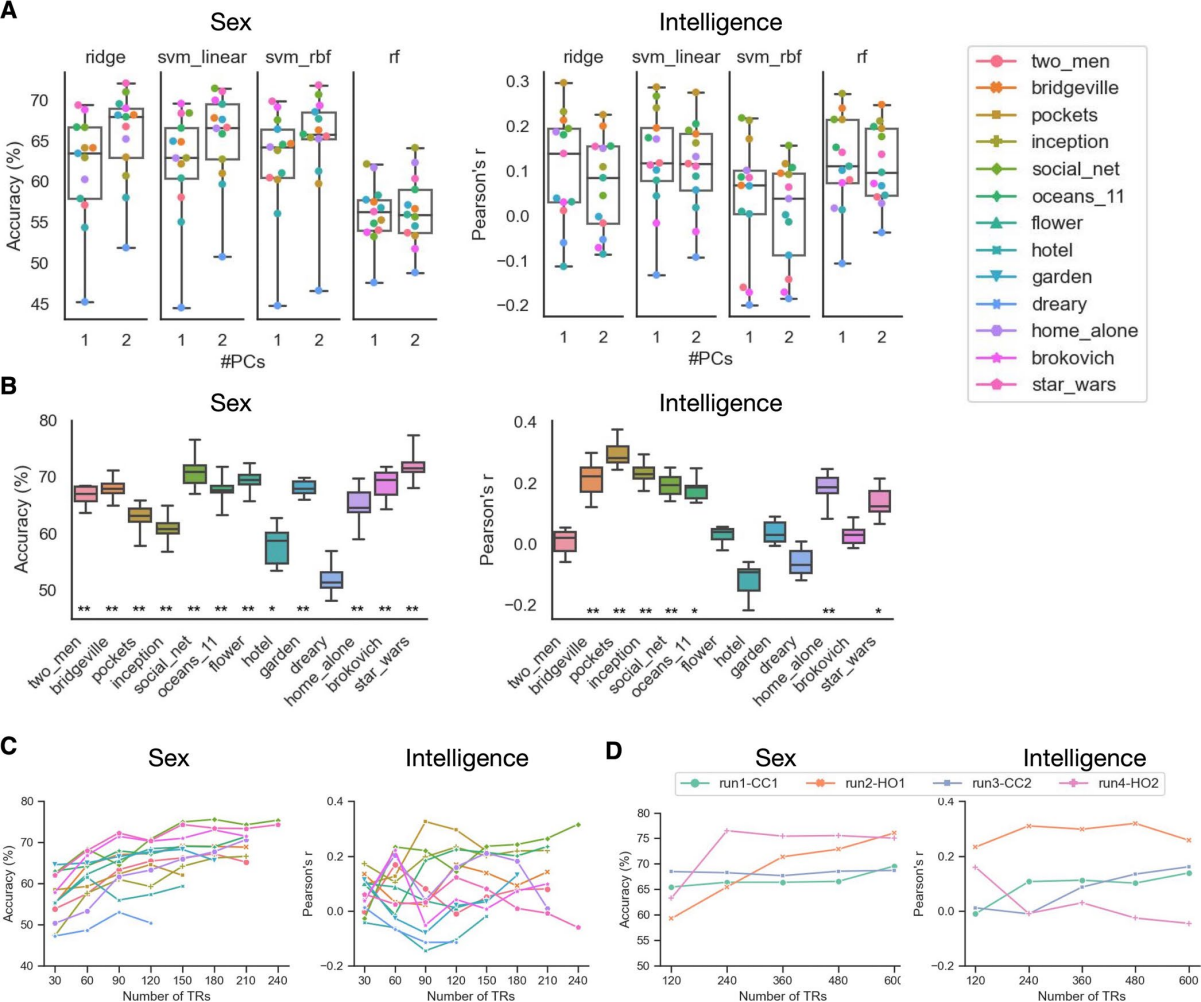
Phenotype prediction performance varies across movie clips. (A) Sex classification accuracy and prediction performance (Pearson's *r* between predicted and observed scores) of fluid intelligence under eight different ML settings. Each dot represents the prediction performance score (averaged over repetitions) under a given setting of a movie clip, with boxplots depicting the distribution over all movie clips (boxplot convention is consistent with that used in Figure 2A). Different movie clips are represented by different colours. #PCs denotes the number of PCs used in prediction for each ROI. (B) Prediction performance under the best ML setting for sex and fluid intelligence separately. The boxplots show the scores from 10 repetitions. A permutation test (1000 iterations) was used to evaluate whether the performance score was significantly above chance. * denotes *p* < 0.05 (FDR corrected). ** denotes *p* < 0.01 (FDR‐corrected). Prediction performance across different data lengths at shorter (from 30 to 240 TRs) (C) and longer time scales (from 120 to 600 TRs) (D) for sex and fluid intelligence separately. Each data point represents the average prediction performance over all repetitions under each condition. Shorter data were obtained by removing extra TRs from the end for each movie clip separately. Longer data were obtained by combining data from multiple movie clips within each of the four movie runs. CC and HO represent the runs with independent films and Hollywood films, respectively.

We tested two feature spaces (PC1 loadings: 436 features; PC1 and PC2 loadings: 872 features) in combination with four different ML algorithms, namely ridge classifier/regression (ridge), support vector machine (SVM) with a linear kernel (svm_linear), SVM with a radial basis function kernel (svm_rbf), and random forest (rf) for phenotype prediction. Figure 4A shows the prediction performance (averaged over all repetitions) of each movie clip under the eight settings for sex and fluid intelligence separately. As expected, we observed substantial differences in achievable accuracy across movie clips for both phenotypes, and the patterns over the clips were largely consistent across different ML settings within each phenotype.

In the following analyses, we focused on the setting achieving the best performance (averaged over movie clips), which is ridge classifier with PC1 and PC2 loadings for sex, and ridge regression with PC1 loadings for fluid intelligence (shown in Figure 4B). For sex classification, accuracies significantly exceeded chance level (permutation‐based *p* < 0.05; FDR corrected) for all movie clips except for “dreary” (51.8%). “Star wars” achieved the highest accuracy (72.0%). For fluid intelligence, 7 out of the 13 movie clips had significant prediction performance (p < 0.05; FDR corrected), with “pockets” achieving the best performance (*r* = 0.30). Significance of the differences between movie clips in prediction performance was confirmed by corrected resampled *t* tests (Figure S3). Further analyses using different data lengths (varying from 30 to 600 TRs) show that large variations in prediction performance across different movie clips and segments existed regardless of the data length, although prediction performance tended to improve as data length increased (Figure 4C,D). In the supplementary analysis, we tested eight additional phenotypes reflecting various aspects of cognitive abilities (working memory, episodic memory, and reading ability) and personality traits (openness, agreeableness, conscientiousness, extraversion and neuroticism) using ridge regression based on PC1 loadings (Figure S4). Among these phenotypes, working memory and reading ability were best predicted overall, with 10 and 6 of the 13 movie clips achieving significant predictions, respectively. For all the phenotypes, consistent with our main findings, we observed substantial differences in predictive performance between movie clips.

#### 2.2.2 Predictive Features and Models

In addition to prediction performance, we examined predictive features and models of the movie clips with significant predictions. We computed the permutation importance for each feature (Breiman 2001), quantified as the decrease in prediction performance score when a feature was shuffled across subjects. Features obtaining a positive importance value (averaged over 1000 iterations) were identified as predictive features (see Figures S5 and S6 for permutation importance maps of individual movie clips).

**FIGURE 5:**
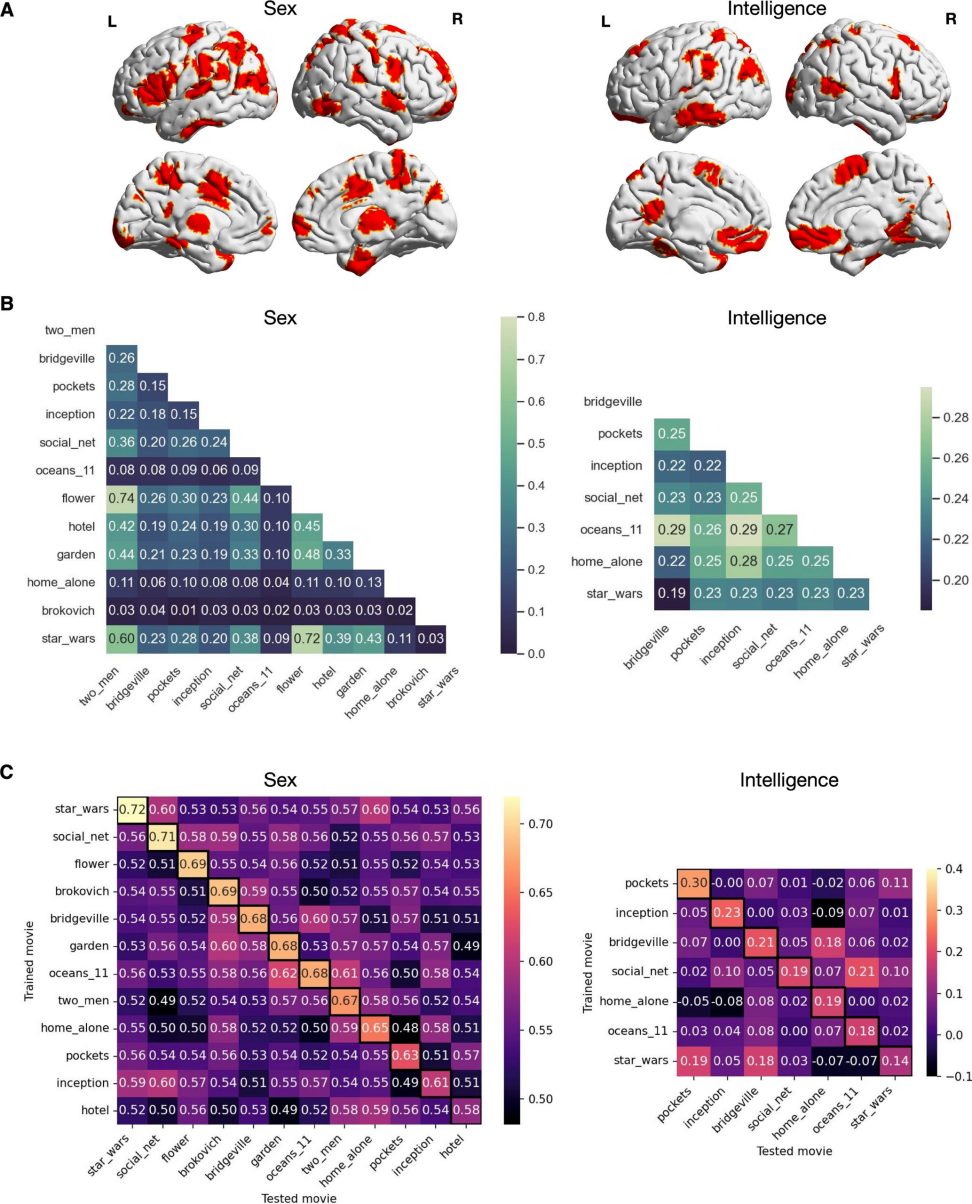
Predictive features and cross‐movie generalisability of predictive models. (A) Predictive features for sex classification and prediction of fluid intelligence separately. Only ROIs that were consistently identified as predictive features across more than half of the analysed movie clips are shown here. (B) Jaccard similarity of predictive features between each pair of movie clips for each phenotype separately. Higher similarity values are indicated by lighter colours. Note that for sex classification only the result based on PC1 loadings is shown here. The result based on PC2 was highly similar to that based on PC1 (Figure S7). (C) Cross‐movie generalisability of predictive models for each phenotype separately. Movie clips are ordered according to their within‐movie prediction performance (diagonals marked in boxes). Better predictions are indicated by lighter colours.

Figure 5A shows ROIs that were consistently identified as predictive features across more than half of the analysed movie clips for sex and fluid intelligence separately. For sex classification, the predictive features involved language, emotion and memory processing systems, where sex differences have been consistently reported by resting‐state studies (Weis et al. 2020; Zhang et al. 2018). In addition, we found that many predictive features came from the dorsal and ventral attention networks, where sex differences have been observed during various attention tasks (Bayliss, di Pellegrino, and Tipper 2005; Cárdenas, Harris, and Becker 2013). For fluid intelligence, we found that most predictive features fell in FPN, default mode network (DMN), visual cortices and attention network, which is highly consistent with prior literature (Dubois et al. 2018; Hearne, Mattingley, and Cocchi 2016).

To investigate whether predictive models can generalise across movie clips, we first computed the Jaccard similarity of the predictive features between each pair of movie clips for each phenotype (Figure 5B). Overall, the similarity between clips was low both for sex (average: 0.20 ± 0.17) and for fluid intelligence (average: 0.24 ± 0.03). Note that for “flower,” “star wars,” and “two men” in sex classification, over 300 ROIs were identified as predictive features, resulting in high similarity values between the three movie clips. In a second analysis, we evaluated the cross‐movie prediction performance of the predictive models for each phenotype (Figure 5C). Consistent with the result of predictive features, the cross‐movie prediction performance was overall low both for sex (average accuracy: 0.55 ± 0.03) and fluid intelligence (average *r* = 0.04 ± 0.07). The predictive models generalised only between a few movie clip pairs, with the highest prediction score achieving 0.62 for sex (“oceans_11” to “garden”) and 0.21 for fluid intelligence (“social_net” to “oceans_11”). Together, these results show that predictive features and models can be highly specific to the used movie clip, and different movie clips can highlight distinct aspects of the same brain–behaviour relationship.

### 2.3 Influence of Inter‐Subject Synchrony on Phenotype Prediction

**FIGURE 6:**
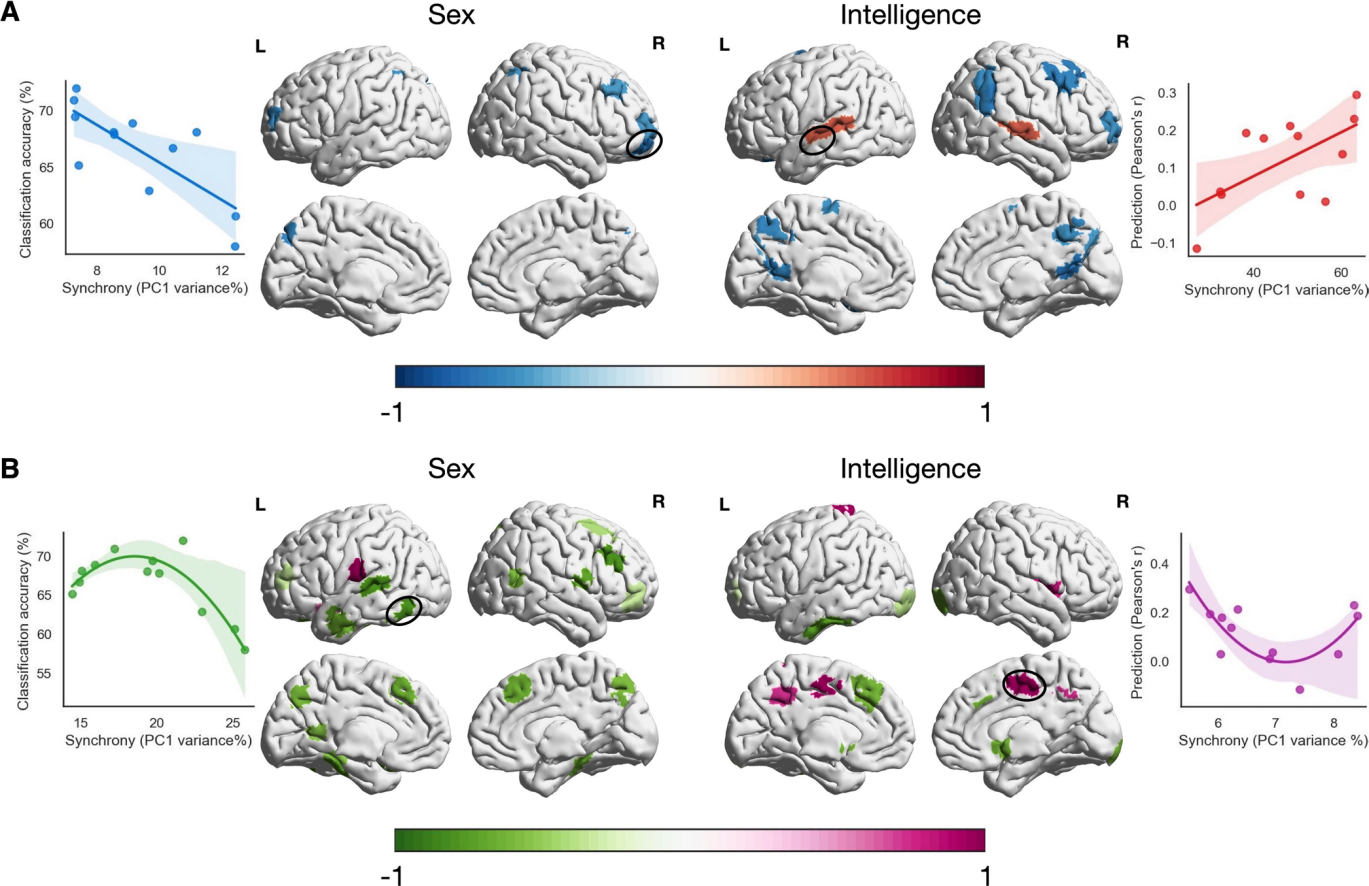
Influence of inter‐subject synchrony on phenotype prediction performance. (A) Linear effect of synchrony on predictions. Brain regions exhibiting a linear effect of synchrony on prediction performance are shown here for each phenotype. Blue and red colours indicate negative and positive regression coefficients of the linear term, respectively. (B) Quadratic effect of synchrony on predictions. Brain regions exhibiting a quadratic effect of synchrony on prediction performance are shown here for each phenotype. Green and violet colours indicate negative and positive regression coefficients of the second‐order term, respectively. A scatter plot between inter‐subject synchrony and prediction performance scores is shown for four representative brain regions (marked by black circles), with each dot representing a movie clip.

Neural synchronisation across subjects is a fundamental phenomenon and also an important basis for us to understand complex brain functions in naturalistic conditions (Hasson et al. 2004; Nastase et al. 2019). Group‐level studies often favour stimuli maximising inter‐subject synchrony for enhanced signals of interest. However, considerations of synchrony for studies on individual differences may be different, as stronger synchrony also means less inter‐subject variability. Then how does the evoked inter‐subject synchrony influence phenotype predictions in naturalistic conditions? To address this question, we explicitly modelled the relationship between inter‐subject synchrony and prediction performance across movie clips using linear regression. Specifically, for each ROI, we tested the linear and quadratic effects of synchrony on prediction performance scores across movie clips by fitting two separate models. Note that we excluded “dreary” from further analyses to avoid obtaining effects that are mainly driven by this “outlier” clip. Bayesian information criterion (BIC), which balances the goodness of fit and model complexity, was used to select the better model (i.e., the one with the lower BIC score) for each ROI. Figure 6 shows coefficients of the corresponding linear or quadratic term in the better model (coefficients of non‐significant models are set to zero) for sex and fluid intelligence separately. The relationship between synchrony and prediction performance across clips falls into the following four types.

Negative linear effect (blue): better predictions are associated with lower inter‐subject synchrony. For both phenotypes, ROIs with such an effect mainly came from the FPN, such as prefrontal cortex, inferior parietal lobe (IPL) and precuneus. This is consistent with previous findings that the FPN network plays an important role in individual identification (Finn et al. 2015; Vanderwal et al. 2017).

Positive linear effect (red): better predictions are associated with higher inter‐subject synchrony. We found such an effect in bilateral STG and superior temporal sulcus (STS) for fluid intelligence. These brain regions are responsible for general processing of movie stimuli, such as multisensory integration and social perception (Beauchamp et al. 2004; Lahnakoski et al. 2012).

Negative quadratic effect (green): prediction performance peaks when synchrony reaches a medium level. We found that such an effect was highlighted in the DMN for sex, and located in the extrastriate visual cortex, prefrontal and inferior temporal gyrus for fluid intelligence. All these brain regions are closely related to the processing of audiovisual stimuli (Khosla et al. 2021; Simony et al. 2016; Sugihara et al. 2006) while being important predictive features for the respective phenotype (Dubois et al. 2018; Hearne, Mattingley, and Cocchi 2016; Weis et al. 2020).

Positive quadratic effect (violet): good prediction performance can be achieved when synchrony is either low or high. We found that ROIs with such an effect mainly came from the sensory‐motor network for both phenotypes, including insular, postcentral gyrus, supplementary motor cortex, and precentral gyrus. These brain regions have been shown to be active when subjects observe actions and touch (Keysers and Gazzola 2009) and involve emotion processing, such as emotion arousal (Nummenmaa et al. 2012) and empathy (Borja Jimenez et al. 2020).

In the supplementary analysis, we performed the same analysis for working memory and reading ability—the two additional phenotypes that were best predicted by the movie clips overall (Figure S8). While each phenotype exhibited a distinct pattern, results on working memory and reading ability were largely consistent with our main findings on sex and fluid intelligence.

### 2.4 Influence of Movie Features on Phenotype Prediction

#### 2.4.1 Hollywood Versus Independent Movie Clips

**FIGURE 7:**
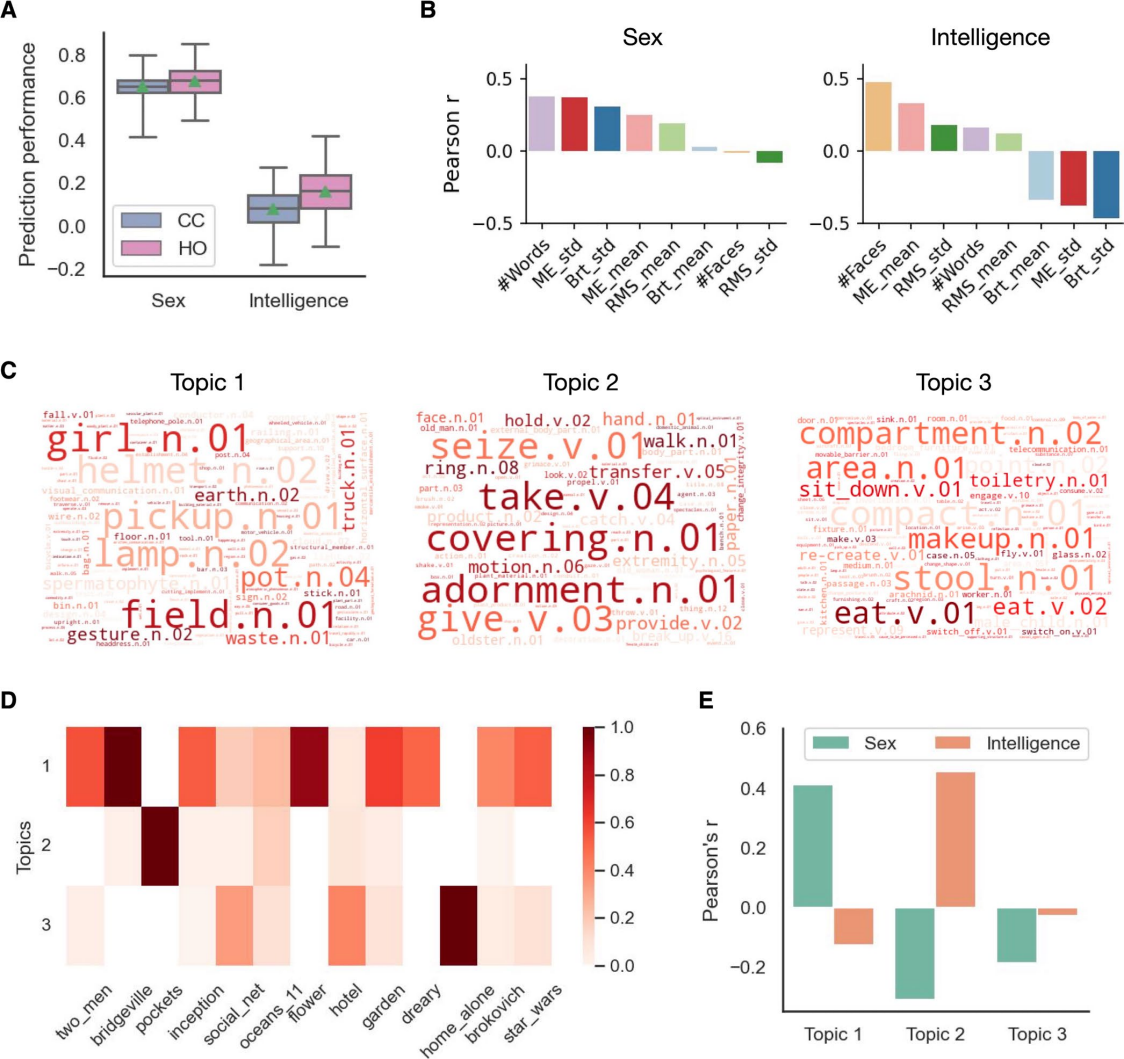
Influence of multi‐level features of movie stimuli on phenotype prediction performance. (A) Comparison between independent (CC) and Hollywood (HO) movie clips in phenotype prediction performance. Prediction performance scores were averaged over movie clips within each film group for each CV fold separately. The boxes show these scores over all 100 CV folds. Boxplot convention is consistent with that used in Figure 2A, with the green triangles indicating the mean. (B) Correlations (Pearson's *r*) between movie features and phenotype prediction performance across movie clips. The correlation is computed for each movie feature and phenotype separately. (C) Semantic topics derived from the matrix of appearing frequency of semantic labels of all movie clips by using NMF, represented in word clouds. The size of each word is proportional to its weight in the corresponding topic. (D) Loadings of each semantic topic in (C) onto each movie clip. (E) Correlations (Pearson's *r*) between prediction performance and loadings of each semantic topic across movie clips for each phenotype separately.

We next went to the movie stimuli themselves to understand how features from different levels of the movie clips influence their predictive capability for phenotypes. First, we asked whether prediction performance differed between Hollywood and independent films (Figures 7A and S9A). Prediction performance scores were averaged over movie clips within each film type for each CV fold. We found that Hollywood movie clips achieved significantly better prediction performance than independent movie clips only for fluid intelligence (*p* = 0.049; corrected resampled *t* test) but not for the other phenotypes (sex: *p* = 0.32, working memory: *p* = 0.69, reading ability: *p* = 0.20).

#### 2.4.2 Low‐ and Middle‐Level Movie Features

To characterise basic motion, visual, and audio properties of movie stimuli, we computed the mean and standard deviation over TRs of total motion energy (ME_mean, ME_std) (Nishimoto et al. 2011), visual brightness (Brt_mean, Brt_std), and audio root‐mean‐square (i.e., loudness; RMS_mean, RMS_std) for each movie clip. We further quantified the social content of each movie clip by computing the total number of TRs showing human faces (#Faces) and the total number of spoken words (#Words). This resulted in eight movie features (Table S2). To understand their influence on prediction performance, we correlated the value of each feature with prediction performance scores across movie clips for each phenotype (Figures 7B and S9B). Overall, better prediction performance was related to increasing social content. Specifically, sex classification was most closely related to #Words (*r* = 0.38), whereas prediction for fluid intelligence was most closely related to #Faces (*r* = 0.48). This aligns with our earlier finding on predictive features: language‐related brain regions (e.g., left STG) (Mesgarani et al. 2014) were prominent in sex classification whereas face‐sensitive brain regions (e.g., posterior STS) (Allison et al. 2000) were highlighted for fluid intelligence.

Furthermore, we found that better sex classification performance was associated with large variations in visual and motion features across time (ME_std and Brt_std), suggestive of diverse and dynamic visual content of the movie clips. This is in line with previous work observing substantial sex differences in visual motion processing (Murray et al. 2018). By contrast, these visual and motion features were negatively correlated with predictions of fluid intelligence. Our further analysis showed that these features had strong negative correlations with face content (Figure S10).

Similarly, better predictions for working memory were associated with both low‐level features (e.g., stronger motion energy and loudness) and social features (e.g., more faces and words), while better predictions for reading ability were mainly related to more speech content (Figure S9B). These results reinforce our earlier observations that prediction performance improves with increasing social content, while the most influential features vary across phenotypes.

#### 2.4.3 High‐Level Semantic Features

Last, we examined the relationship between high‐level semantic features and prediction performance. Specifically, we computed the appearing frequency of each semantic label for each movie clip (Huth et al. 2012), resulting in a 297 (semantic labels) by 13 (movie clips) frequency matrix. To reduce data dimensionality and facilitate understanding of the semantic labels, we identified 3 semantic topics (Figure 7C) with their loadings onto each movie clip (Figure 7D), by applying non‐negative matrix factorisation (NMF) to the matrix. Topic 1 highlighted a variety of nouns, mainly concrete objects and concepts such as “girl” and “field,” and was expressed in most of the movie clips. Topic 2 highlighted verbs and human‐related nouns, such as “seize,” “take,” “hand,” and “face.” This topic was mainly loaded onto the movie clip “pockets,” where people are explaining personal meanings of the stuff they keep in their pockets. Topic 3 was heavily loaded onto the Hollywood movie clip “home alone,” which contained many daily life scenarios and household items, such as “stool,” “eat,” and “makeup.” For each semantic topic, we correlated its loading values with prediction performance across movie clips for each phenotype (Figures 7E and S9C). We found that all phenotypes were differentially related to these semantic topics. For example, better sex classification and working memory predictions were associated with higher loadings of topic 1 (reflecting rich visual content). In contrast, better predictions of fluid intelligence were associated with higher loadings of topic 2 (reflecting rich social content). This aligns with our previous findings on the low‐level visual features that better sex classification and working memory predictions favoured dynamic visual motion features, whereas fluid intelligence emphasised visual social content (e.g., #Faces).

### 2.5 Additional Validation Analyses

Head motion has been thought to be a major confounding factor for fMRI studies. To understand the influence of head motion on the differences in predictive capability across movie clips, we quantified head motion as mean framewise displacement (FD) across TRs for each movie clip. We found that movie clips presented later within each run incited stronger head motion (averaged over subjects) relative to those presented earlier (Figure S11A). Such an increase in head motion over time may reflect subject discomfort or fatigue due to long scan duration. Notably, variations in head motion across movie clips did not explain their differences in prediction performance (sex: *r* = 0.25, *p* = 0.41; fluid intelligence: *r* = 0.16, *p* = 0.61; Figure S11B).

We also explored how subject engagement and attention during the movie clips may influence their predictive capability using eye tracking data. Blink rate inhibition has been thought to be associated with enhanced vigilance and attention allocation (Demiral et al. 2023; Maffei and Angrilli 2019). Thus, we calculated blink rate (i.e., the number of blinks divided by the total number of TRs) for each movie clip and subject and examined its relationship with prediction performance across movie clips (Figure S12). As expected, we found that better prediction performance was associated with lower blink rate (averaged across subjects), suggesting higher levels of attention and engagement (sex: *r* = −0.48, *p* = 0.09; fluid intelligence: *r* = −0.57, *p* = 0.04). We also evaluated two additional eye movement indexes—the number of fixations and the peak velocity of saccades—but found no significant relationships with prediction performance (Figure S13). Future work can further investigate the extent to which various eye movement measures reflect subject engagement and attention levels and how these factors influence phenotype prediction.

## 3 Discussion

In this study, we examined how the choice of movie clips influences the study of individual differences and phenotype prediction. First, we examined the inter‐subject variability/synchrony in brain activity during watching different movie clips. We found that compared to low‐level brain regions related to general audiovisual information processing, higher‐order brain regions exhibited larger inter‐subject variability and cross‐movie variability. Second, we investigated differences between movie clips in their performance for phenotype prediction, as well as predictive models and features. In particular, we investigated how inter‐subject synchrony of brain activity influenced prediction performance across movie clips. We found that predictions to a certain degree can benefit from enhanced inter‐subject synchrony in many brain regions. Finally, we examined the influence of various features of movie clips on prediction performance. We found that better predictions were in general associated with rich social content, but the specific influential features differed across phenotypes.

### 3.1 Individual Differences in Brain Activity Evoked During Naturalistic Viewing

How do different movie stimuli influence inter‐subject variability in brain activity? We observed a consistent pattern across movie clips that higher‐order brain regions exhibited larger inter‐subject variability than the audiovisual sensory cortices. Previous studies have shown that low‐level sensory brain regions closely track the external movie stimuli and respond similarly across subjects (Hasson et al. 2004; Hasson, Yang et al. 2008). By contrast, prefrontal and parietal cortices have been shown to reflect personal endogenous processes, such as affective experience (Chang et al. 2021) and autobiographical memory (Anderson et al. 2020). These brain regions have also been shown to exhibit higher inter‐subject variability than the other networks in functional connectivity (FC) profiles (Finn et al. 2015; Mueller et al. 2013). Moreover, the hierarchy in inter‐subject variability of brain activity we observed in this present study aligns well with the sensory‐to‐association gradient axis, which has been thought to constitute a fundamental principle of cortical functional organisation (Margulies et al. 2016). A recent study has further observed such an axis, anchored by auditory and visual regions at one end and transmodal regions such as FPN, DMN, and limbics at the other end, in naturalistic viewing conditions (Samara et al. 2023).

In addition, we found that the stability of the pattern of inter‐subject variability across movie clips also followed such a functional hierarchy. Specifically, the pattern of inter‐subject variability was more similar across movie clips in audiovisual sensory and association brain regions than in higher‐order brain regions. This result suggests that individual differences in activity of the former brain regions may reflect stable personal characteristics in general processing of movie stimuli, such as sensory integration and social perception (Hasson, Landesman et al. 2008; Lahnakoski et al. 2012; Nummenmaa et al. 2012). Our finding is consistent with a previous study identifying similar “trait‐like” brain regions using a different movie watching dataset (Gao et al. 2020). Moreover, individual FC patterns have been found to be highly sensitive to the specific movie content in higher‐order functional systems, such as semantic memory, social cognition and emotion‐related networks (Chen et al. 2020; Kröll et al. 2023), consistent with our findings.

Overall, our results demonstrate that inter‐subject variability in brain activity and its variations across movie clips generally increase along the functional hierarchy of the cortical cortex. Higher‐order brain regions tend to produce less synchronous (i.e., more idiosyncratic) brain activity across subjects and their activity is more variable across movie clips, compared to low‐level audiovisual sensory regions.

### 3.2 Selection of Naturalistic Stimuli Is Important for Studies on Phenotype Prediction

Does the choice of stimulus matter for studies of brain–behaviour relationships with phenotype prediction? By conducting a series of prediction analyses for multiple phenotypes, we demonstrate that movie clips with different contents can lead to robust differences in their predictive capability for phenotypes. Consistent with our results, previous studies have also reported differences in phenotype prediction performance across paradigms under both naturalistic viewing (Finn and Bandettini 2021; Li et al. 2023) and task conditions (Finn et al. 2017; Greene et al. 2018). We further extended these findings by showing that each movie clip had its own unique pattern of predictive features, despite some shared predictive features, and predictive models did not generalise well across movie clips. These results suggest that different movie clips may highlight distinct aspects of individual differences related to the same phenotype. This finding is also in accordance with our previous results that the pattern of individual differences in brain activity was different across movie clips.

Notably, another study using the same dataset for sex classification found that individual FC profiles as well as FC‐based predictive models are highly similar across different naturalistic conditions (Tian et al. 2021). Such inconsistency may arise from the distinction in the used brain features between this study and our present study (FC vs. task‐evoked activity); a previous study on conventional tasks has found that while the network organisation of the brain (i.e., FC profile) stays largely stable across different tasks, the evoked activations highly depend on the specific task (Gratton et al. 2018). Furthermore, different from (Tian et al. 2021), we focused on very short movie clips (~2 min) rather than data from long scan duration (~10 min). In fact, a recent study using the same set of short movie clips has also observed substantial differences in predictions across movie clips based on FC profiles (Finn and Bandettini 2021). When increasing data length, we found that although prediction performance increased in general due to improved signal‐to‐noise ratio, differences across conditions were preserved.

In addition, the movie clip achieving the best prediction performance differed between phenotypes. For example, sex was best classified by “star wars” and intelligence was best predicted by “pockets,” suggesting that these two clips may highlight individual differences related to different phenotypes. “Star wars” elicited brain activity showing sex differences in many functional systems, with predictive features spreading across the whole brain (Figure S5). By contrast, “pockets” highlighted intelligence‐related individual differences in brain activity, with predictive features clustering in the DMN and temporal cortex (Dubois et al. 2018; Hearne, Mattingley, and Cocchi 2016) (Figure S6). These results suggest that the decision on which movie stimulus should be used for phenotype prediction needs to be made specifically for individual phenotypes.

Notably, the non‐social movie clip “dreary” stood out again as the only movie clip that did not significantly predict both phenotypes. A possible explanation is that the low level of evoked synchrony across subjects of “dreary” (Figure 2) hinders the utility of TOPF or other ISC‐based approaches. However, such bad performance of “dreary” seems not specific to the methods we chose in this study. A previous study has also obtained low performance on “dreary” for predicting different phenotypes using FC‐based features (Finn and Bandettini 2021). These findings suggest that “dreary” may not elicit sufficient behaviourally relevant individual differences in brain activity and thus may not be the optimal choice relative to typical social movie clips for phenotype prediction, at least for certain phenotypes.

Altogether, our results highlight the necessity of a careful selection of naturalistic stimuli for studying individual differences and phenotype prediction. Movie stimuli that bring out brain signals related to a specific phenotype tend to facilitate prediction of the phenotype. Stimuli with different contents may highlight distinct aspects of the same brain–behaviour relationship.

### 3.3 Phenotype Prediction Benefits From Enhanced Synchrony in Certain Brain Regions

How does synchrony in brain activity across subjects elicited during a movie clip influence its capability for phenotype prediction? Inter‐subject synchrony is an important consideration for stimulus selection in naturalistic studies (Finn et al. 2020). While group‐level studies tend to maximise synchrony, one might think that low synchrony benefits phenotype prediction where our interest is individual differences. Yet, several previous studies have found that task and naturalistic conditions that make brain states more similar across subjects tend to yield more stable and identifiable individual differences (Finn et al. 2017; Vanderwal et al. 2017). Our results in the present study show that the relationship between synchrony and predictions highly depends on the brain region and phenotype under investigation.

Specifically, lower synchrony (i.e., higher inter‐subject variability) in FPN is associated with better predictions. FPN supports various executive brain functions such as selective attention and working memory (Diamond 2013) and has been thought to reflect the most identifiable information of individuals (Finn et al. 2015; Vanderwal et al. 2017). Movie clips eliciting higher inter‐subject variability in FPN may thus improve predictions by enhancing behaviourally relevant signals of individual subjects. A recent study has also shown that FPN activity reduces distracting thoughts during movie watching (Wallace et al. 2024). By contrast, better predictions can be linked to either low or high synchrony in the sensory‐motor network. The sensory‐motor network has been shown to support emotional arousal and empathy (Borja Jimenez et al. 2020; Nummenmaa et al. 2012; Paracampo et al. 2017). High synchrony in the sensory‐motor network may thus indicate that subjects have high arousal level and emotionally resonate with characters in movie clips, while low synchrony may reflect meaningful individual differences in emotional sensations. This result suggests two divergent ways of how synchrony in sensorimotor regions promotes predictions of individual phenotypes.

Moreover, phenotype prediction benefits from stronger synchrony in brain regions related to lower‐level processing of the movie stimulus. Increased synchrony has been shown to indicate better subject engagement and higher attention levels (Ki, Kelly, and Parra 2016; Ohad and Yeshurun 2023). Movie clips eliciting higher synchrony in corresponding brain regions may lead to reduced task‐irrelevant noises (e.g., caused by tiredness or mind wandering), thus improving signal‐to‐noise ratio for phenotype prediction. However, if a brain region not only supports the general processing of audiovisual stimuli but also relates to the phenotype under investigation, then a medium level of synchrony may be optimal for prediction of this phenotype. For such brain regions, both a strong enough shared response of stimulus processing across subjects and adequate individual differences are necessary. Similarly, a recent study has found that phenotype prediction on task fMRI data also benefits from a medium level of inter‐subject synchrony (Greene et al. 2020).

Overall, how synchrony influences predictions in a brain region is related to its functional hierarchy. For higher‐order brain regions where subjects often have idiosyncratic activity, better predictions are associated with lower synchrony. For low‐level brain regions that often produce synchronous activity across subjects, better predictions benefit from higher synchrony. Our results provide novel and important insights into how inter‐subject synchrony of brain activity influences predictions of individual phenotypes for future research.

### 3.4 Appreciation of Complex Movie Features Facilitates Stimulus Selection for Phenotype Prediction

What features make a movie stimulus useful for phenotype prediction? We gained several empirical insights into this question by analysing a variety of movie features at different levels. First, Hollywood movie clips do not necessarily outperform other types of movie clips for phenotype prediction. Hollywood movies are believed to be easier to understand and appealing to broader audiences than independent movies. They often use conventional storytelling techniques (e.g., clear protagonists and antagonists and linear plotlines) with advanced cinematography techniques to facilitate storytelling (Bordwell 2006; Hasson, Landesman et al. 2008; Smith, Levin, and Cutting 2012). Besides, they are more likely to have been seen by subjects before scanning relative to independent movies. Such features allow Hollywood movie clips to better captivate subjects (Chen et al. 2017; Jääskeläinen et al. 2008) and thus reduce noises for phenotype prediction. This is consistent with our results that Hollywood movie clips on average achieved better prediction performance than independent movie clips. However, such differences in prediction performance between the two film types were only significant for fluid intelligence but not for the other phenotypes. Besides, the movie clip with the best performance for fluid intelligence was not from a Hollywood film. These results suggest that there are also other factors influencing the predictive capability of a movie stimulus apart from its film type.

Second, our analysis on various visual, auditory and semantic features show that better predictions were associated with rich social content. Social processing of naturalistic stimuli has been previously found to enhance activity in various higher‐order brain functions, such as episodic memory formation (Hasson, Furman et al. 2008) and emotion processing (Nummenmaa et al. 2012). In line with our results, prior studies have observed similar effects of social content on the reliability of individual differences and phenotype prediction performance (Finn and Bandettini 2021; Gao et al. 2020).

Third, the specific influential movie features vary across phenotypes. We found that better sex classification accuracy was associated with rich language and visual content. These features may allow the movie stimulus to bring out meaningful sex differences in various brain functions, such as language, emotion or visual processing (Baxter et al. 2003; Kret and De Gelder 2012; Murray et al. 2018). By contrast, prediction of fluid intelligence was associated with images of human faces and social interactions. This finding is consistent with studies that have shown the relationship of intelligence with face processing and social cognition abilities (Ibanez et al. 2013; Walker et al. 2023).

Overall, movie features at various levels can influence the predictive capability of movie clips. While social content in general promotes predictions, different phenotypes may favour distinct specific movie features. Therefore, our choice of movie stimulus should be made for individual phenotypes specifically and may be further optimised by a more comprehensive and deeper understanding of movie features.

### 3.5 Considerations in Stimulus Selection for Phenotype Prediction

In summary, our study offers valuable insights into selecting appropriate stimuli for phenotype prediction in naturalistic conditions. First, stimulus selection is crucial for studies on individual differences and prediction of individual phenotypes across various domains, including sex, cognition and personality traits. Second, inter‐subject synchrony of brain activity emerges as a promising tool for stimulus selection. Strong synchrony in audiovisual sensory and associated brain regions, low synchrony in higher‐order brain regions, and moderate synchrony in brain regions involved in both general stimulus processing and the target phenotype may enhance prediction performance. Third, compared to non‐social movie clips, clips with rich social content generally improves phenotype prediction.

More importantly, there may not be a single stimulus suitable for all research questions, as our results demonstrated that different phenotypes were best predicted by movie clips with distinct content and features. Therefore, selecting specific stimulus features and content should be tailored to each research question. For instance, in our study, sex was best classified by “star wars,” a clip characterised by dynamic visual input (e.g., the sudden appearance of a beast) and socially and emotionally charged scenes (e.g., a woman and man arguing). These features likely highlight sex differences in interpretations and experiences, engaging visual, emotional and language processing systems (Codispoti, Surcinelli, and Baldaro 2008). Fluid intelligence, in contrast, was best predicted by “pockets,” a clip featuring personal stories and close‐up shots of faces and hand movements. Such characteristics may amplify individual differences related to intelligence in face and social processing (Ibanez et al. 2013; Walker et al. 2023). Similarly, working memory was best predicted by “inception,” a Hollywood clip set in an imaginary world with rich social and motion information and high loudness levels (Table S2). These features may maintain high levels of attention and engagement in subjects, consistent with our result that this clip elicited the highest level of inter‐subject synchrony (Figure 2). This may enhance the prediction of working memory as attention and working memory have been shown to share common neural substrates (Mayer et al. 2007). By comparison, reading ability was best predicted by “garden,” a documentary clip featuring a woman presenting a gardening program. This clip contains the highest speech content among all clips, which may strongly engage the language processing system and thus suit the study of reading abilities. Taken together, an appropriate stimulus may need to be chosen in a way as to elicit the brain into a state that effectively engages the functional systems underlying the phenotype under investigation.

Apart from the phenotype of interest, participants are another crucial consideration in stimulus selection. One of the key advantages of naturalistic viewing over resting and conventional task conditions is its applicability to clinical and other populations that may struggle with task compliance during scanning, such as staying awake or minimising head movement (Eickhoff, Milham, and Vanderwal 2020; Vanderwal, Eilbott, and Castellanos 2019). Special care is needed when choosing stimuli for these populations. For example, movie segments with less emotional content have been shown to better reveal the relationship between brain responses and depressive symptoms in adolescence compared to emotional segments (Gruskin, Rosenberg, and Holmes 2020). Additionally, age significantly shapes individual differences in brain responses during movie watching, with distinct preferences for stimuli across age groups (Di et al. 2023; Geerligs and Campbell 2018). Factors such as participants' familiarity with the stimulus, personal engagement, and movie watching habits and preferences may also strongly influence brain responses (Andric et al. 2016). Future work is needed to understand how these factors influence phenotype prediction to optimise stimulus selection. In sum, considerable work is still needed to achieve the ultimate goal of selecting an appropriate stimulus for each research question.

### 3.6 Future Directions

Our present study focuses on brain features derived from evoked brain activity for phenotype prediction, as the evoked activity is of most behavioural relevance in naturalistic conditions. However, there is also great interest in using FC‐based features for prediction (Greene et al. 2022). While we observed convergence between our findings and prior studies using FC‐based features (Finn and Bandettini 2021), a systematic evaluation of the influence of stimulus selection on FC‐based prediction will still be of interest to future studies. In addition, in this study, individual differences in brain activity were characterised at the ROI level rather than the voxel level. This approach was necessary for our prediction analysis, as brain parcellations offer a biologically meaningful way to reduce the high dimensionality of voxel‐wise fMRI data. Future studies could further validate our findings by employing different brain parcellations or using other dimensionality reduction techniques, such as functional alignment (Chen et al. 2015; Haxby et al. 2011). Moreover, our analysis on the influence of inter‐subject synchrony and movie features on phenotype prediction across movie clips may be constrained by the limited number of clips. Nonetheless, our findings remain largely consistent across different phenotypes. Future work could validate these findings using datasets with a larger number of movie clips and greater content diversity. Finally, future work could deepen our understanding of stimulus selection by incorporating more detailed movie annotations (e.g., emotion), collecting additional subject information (e.g., familiarity with clips and film preferences), and ensuring a balanced presentation order of clips to better control for subjects' tiredness and arousal levels.

In summary, we demonstrate that the choice of stimulus in naturalistic conditions can significantly influence the level and pattern of inter‐subject variability in brain activity, the performance for predicting individual phenotypes, and the learned brain–behaviour relationship. Importantly, we demonstrate the possibility of selecting an appropriate stimulus for a specific phenotype according to the level of synchrony in brain activity across subjects and features of the stimulus. Our results underscore the significance of stimulus selection and provide timely and important insights into how the choice of stimulus can influence downstream analysis. This can in turn facilitate future investigations to leverage the full potential of naturalistic conditions for studying individual differences and brain–behaviour relationships.

## 4 Methods

### 4.1 Participants

We considered all available subjects in the HCP study (S1200 release) who participated in movie watching scanning sessions (Van Essen et al. 2013). Six subjects were excluded from further analyses due to not completing all movie watching tasks, yielding 178 subjects (108 females, age = 29.40 ± 3.31) from 90 unique families. The HCP study was approved by the Washington University institutional review board. Informed consent was obtained from all participants.

### 4.2 Naturalistic Stimuli

The HCP study comprised two movie watching scanning sessions, each session containing two separate runs of a length of roughly 15 min. During each run, three to four short different movie clips (1–4 min in length) were presented with a rest block of 20s as an interval between movie clips. At the end of each run, a repeat validation clip was presented. The validation clip as well as the shortest clip “overcome” (1′03″) were excluded, resulting in 13 movie clips in our analyses. Each of the 13 movie clips was presented only once during scanning, with the length varying from 2′22″ to 4′19″. Movie clips in runs 1 and 3 were from independent (CC) films, whereas those in runs 2 and 4 were from Hollywood (HO) movies. A brief description of the content and length of these movie clips are provided in Table S1. All the movie stimuli can be downloaded from the HCP website (https://db.humanconnectome.org/).

### 4.3 Imaging Data Acquisition and Processing

All fMRI images during movie watching were acquired at a 7 T Siemens scanner (TR = 1000 ms, TE = 22.2 ms, resolution = 1.6 mm^3^). We used the fMRI data preprocessed by the standard HCP pipeline, including steps of motion correction, registration to the standard MNI space, high‐pass temporal filtering, 24 motion parameters removal and FIX denoising (Glasser et al. 2013). The preprocessed data were available from the HCP website. No subjects were further excluded based on the exclusion criterion of having a mean FD larger than 0.5 mm (Power et al. 2014). Data during rest blocks were discarded, so that only data during watching movie clips were used. We further removed the first 10 volumes of each movie clip to avoid unstable signals. A whole‐brain parcellation, containing 400 cortical (Schaefer et al. 2018) and 36 subcortical ROIs (Fan et al. 2016), was applied to the remaining data. A mean time series over voxels was extracted for each ROI, subject, and movie clip, using the data processing and analysis for brain imaging (DPABI) toolbox (Yan et al. 2016) (http://rfmri.org/dpabi). For a fair comparison across movie clips, all the resulting time series were truncated to 132 TRs, that is, the length of the shortest clip, “dreary,” by removing the extra TRs at the end of each clip.

### 4.4 Eye Tracking Data Acquisition and Processing

Eye tracking data were provided by the HCP and collected using an Eyelink S1000 system with two different sampling rates (1000 and 500 Hz). In this study, we only analysed data from subjects with a sampling rate of 1000 Hz for a fair comparison. Subjects were further excluded if their data were unavailable for each movie run. Such information can be obtained from the eye tracking data as well as their metadata provided by the HCP. This resulted in a different number of subjects for each movie run for our analysis, varying from 131 to 135 subjects.

### 4.5 Inter‐Subject Synchrony

To measure inter‐subject variability in brain activity, we computed inter‐subject synchrony, that is, the level of synchronisation of fMRI signals across subjects. For each ROI, we applied a PCA to the z‐score normalised fMRI time series across all subjects within each movie clip. The variance explained by PC1 was used to quantify the inter‐subject synchrony (Li et al. 2023). A greater value of the variance explained by PC1 indicates that subjects were more consistent in their brain activity over time, suggestive of smaller inter‐subject variability. This measure of inter‐subject synchrony has been shown to be very similar to the widely used subject‐averaged inter‐subject correlations (Di and Biswal 2022; Nastase et al. 2019). The spatial pattern of inter‐subject synchrony across the whole brain was compared for each pair of movie clips by using Pearson's correlation, resulting in a 13 by 13 correlation matrix.

### 4.6 Individual Differences and Between‐Movie Variability

Individual differences in neural response to the same stimulus were operationalised as individual subjects' expression levels of a response shared across subjects (Di and Biswal 2022; Finn et al. 2020). In each ROI, the PC1 of the z‐score normalised fMRI time series across subjects was used to represent the shared response. Correspondingly, the subject‐wise PC1 loadings reflected how strongly the shared response was expressed in the observed brain signals of individual subjects. A higher loading value indicates greater resemblance of a subject's brain activity to the shared response. To examine how individual expression levels vary across movie clips, we correlated these PC1 loadings across all subjects between each pair of clips within each ROI, resulting in a 13 by 13 correlation matrix for each ROI.

### 4.7 Phenotype Prediction by TOPF

To understand how stimulus selection influences the utility for phenotype prediction, we investigated two different phenotypes in the main analysis, namely biological sex and fluid intelligence (“PMAT24_A_CR”). Fluid intelligence was measured as the number of correct responses of Raven's progressive matrices (Bilker et al. 2012). In the supplementary analysis, we investigated eight additional phenotypes related to cognition (working memory: “ListSort_Unadj,” reading ability: “ReadEng_Unadj,” and episodic memory: “PicSeq_Unadj”) and personality traits (openness: “NEOFAC_O,” agreeableness: “NEOFAC_A,” conscientiousness: “NEOFAC_C,” extraversion: “NEOFAC_E,” and neuroticism: “NEOFAC_N”). Phenotype prediction was conducted by using our previously proposed method, TOPF (Li et al. 2023). Briefly, the TOPF method applies a PCA to the z‐score normalised fMRI time series across subjects for each ROI, and uses the PC loadings (after z‐score normalisation) from all ROIs as features for prediction in a ML framework.

We tested different ML settings for phenotype prediction. Specifically, we used two different feature spaces, which are PC1 loadings and loadings of both PC1 and PC2 of all ROIs, resulting in 436 and 872 features, respectively. Note that no further PCs were tested because we have previously found on the same dataset that only the first two PCs captured a statistically significant amount of shared variance across subjects (Li et al. 2023). In addition, we tested four ML algorithms, which are ridge classifier/regression, SVM with a linear kernel, SVM with a radial basis function kernel, and random forest classifiers/regression.

Model performance was evaluated in a nested CV setting. We adopted a 10‐fold CV in the outer loop, where in each fold a predictive model was fitted on the training sample and then used to predict phenotypes of subjects in the test sample. We note that features of test subjects were computed separately from those of training subjects to prevent data leakage. While for training subjects, the PC loadings were used as features, for test subjects the features were computed as the Pearson's correlation coefficients between their respective fMRI time series and the PCs learned on the training sample (Li et al. 2023). We also accounted for the family structure of the subjects by including subjects from the same family in either the training or test set for each fold. Regularisation parameters were optimised via a grid search with inner fivefold CVs.

Sex classification performance was measured by balanced accuracy to avoid overestimation caused by class imbalance. Prediction performance of the other phenotypes was quantified as the Pearson's correlation coefficient between predicted and observed scores. The above procedure was repeated 10 times with different splits of the sample. The whole procedure was performed in Python based on the Julearn packages (https://juaml.github.io/julearn/main/index.html) (Hamdan et al. 2023).

### 4.8 Effect of Data Length on Phenotype Prediction

We conducted two analyses to evaluate the effect of data length on phenotype prediction. First, for each movie clip, we gradually increased data length (N_tr) by steps of 30 TRs (i.e., 30 s) starting from N_tr = 30 until N_tr exceeded the total length of the given movie clip, yielding a maximum of eight different data lengths. We then reapplied the TOPF approach to extract features and perform phenotype prediction for each N_tr and each movie clip separately using the same procedure as described before. Second, we conducted a similar analysis but used a longer time scale by concatenating data across movie clips within each run. We started from N_tr = 120 and increased data length by steps of 120 TRs (i.e., 2 min) until N_tr = 600, resulting in five different data lengths. fMRI data were concatenated in the order of the movie clips as they were presented within each run.

### 4.9 Significance of Prediction Performance and Model Comparison

We applied a permutation test to evaluate whether prediction performance achieved significantly above the chance level for each movie clip. We randomly shuffled the labels (i.e., the phenotypic scores) across subjects 1000 times and for each permutation we rerun the classification/prediction pipeline as was described above and computed a performance score (average over all CV folds). A *p*‐value was then calculated based on a null distribution constructed by these performance scores obtained on permuted data. The *p*‐value was defined as the proportion of permutations where the performance score was higher or equal to the original performance score that was derived from the non‐permuted data. To test whether the difference in prediction performance between two movie clips was significant, we employed corrected resampled paired *t* tests to the accuracies across all 100 CV folds for each pair of movie clips (Nadeau and Bengio 2003). This approach accounted for the fact that CV folds were not independent.

### 4.10 Permutation Feature Importance

We used permutation feature importance to evaluate the importance of each feature for classification/prediction (Breiman 2001). Given a model, we randomly shuffled the values of a feature across subjects in the test data. The feature importance was defined as the decrease in the performance score when applying the model to the shuffled data. For each model and each feature, we shuffled the data 1000 times and calculated the mean feature importance over all iterations. We then averaged the permutation feature importance over all models within each movie clip for each feature. Features with a positive average importance were identified as a predictive feature. Calculation of permutation feature importance was conducted by using the “PermutationImportance” function in the ELI5 python package (https://github.com/eli5‐org/eli5). We used the Jaccard similarity to quantify the similarity in predictive features for each pair of movie clips.

### 4.11 Cross‐Movie Prediction Performance

To investigate the generalisability of predictive models across movie clips, we evaluated the cross‐movie prediction performance for each pair of clips based on the models and features obtained in the previous analysis. Specifically, given a pair of clips A and B, in each fold, we applied the model previously fitted on the training data of clip A to the corresponding test data of clip B. To improve the comparability across clips, the sign of each PC and loading was flipped if the maximum of the corresponding PC loadings was not equal to the maximum of the absolute values of the loadings. Similarly, the cross‐movie prediction performance was computed for each fold and then averaged over all folds of all repetitions. Note that the generalisability from clip A to clip B is normally different from that from clip B to clip A.

### 4.12 Relationship Between Inter‐Subject Synchrony and Phenotype Prediction Performance Across Clips

We built two different linear regression models to examine the influence of inter‐subject synchrony on prediction performance across clips for each phenotype. Note that the only non‐social movie clip “dreary” was excluded from further analyses. The following two models were used to examine the linear and quadratic effects of synchrony on prediction performance scores, respectively:

$$\text{Linear effect}:y=\beta_{0}+\beta_{1}*x+\varepsilon$$

$$\text{Quadratic effect}:y=\beta_{0}+\beta_{1}*x+\beta_{2}*x^{2}+\varepsilon$$

Each model was fitted on prediction performance scores (y) and inter‐subject synchrony (x) across movie clips for each ROI separately, by using the “statsmodels.regression.linear_model.OLS” function (Seabold and Perktold 2010). β represents the regression coefficient of the corresponding term and ε denotes the residual. Z‐score normalisation was applied to x and y before model fitting. BIC was used for model selection, which balances the goodness of fit and model complexity. For each ROI, the model achieving the lower BIC value was identified as the better model.

### 4.13 Comparison Between Clips From Hollywood Films and Independent Films

We tested whether phenotype prediction performance differed between Hollywood (“inception,” “social net,” “ocean 11,” “home alone,” “brockovich,” and “star wars”) and independent movie clips (“two men,” “bridgeville,” “pockets,” “flower,” “hotel,” and “garden”). Specifically, we averaged the prediction performance scores over movie clips within each film group for each CV fold separately, yielding 100 values for each group. A corrected resampled *t* test was used to examine the significance of the difference between the two groups. This analysis was done for each phenotype separately.

### 4.14 Analysis of Low‐ and Middle‐Level Movie Features

A variety of features were extracted for each movie clip to characterise basic properties of the movie stimuli. Specifically, for each TR, we computed total motion energy (Huth et al. 2012), as the mean over all 4025 channels (provided by the HCP) (Nishimoto et al. 2011). We also extracted visual brightness and auditory loudness (the root mean square of audio signals) at each time point. Then, for each of the three measures, we computed the mean and standard deviation (std) of the values across time within each clip, yielding six low‐level features for each clip. To quantify the social content of each movie clip, we extracted two features, that is, the total number of TRs when human faces were shown (#Faces) and the total number of words presented in the audio (#Words). #Faces, brightness and loudness were extracted automatically by using a python‐based feature extraction toolbox, Pliers (McNamara, De La Vega, and Yarkoni 2017). For the computation of #Words, we used a deep learning based‐model for speech recognition provided by Whisper (Radford et al. 2022) to automatically transcribe speech in the audio of each movie clip.

### 4.15 Analysis of High‐Level Semantic Features

High‐level semantic features were derived based on the 859 semantic (WordNet) labels for each image (Huth et al. 2012), provided by the HCP. For each clip, we computed the appearing frequency of each semantic label as the number of TRs showing the label divided by the total number of TRs of the clip (i.e., 132). Labels that had values of no variation across clips were excluded, resulting in 297 features for further analysis. To reduce data dimensionality and facilitate interpretation, we applied NMF (Lee and Seung 1999) to the data matrix of semantic label frequency of all movie clips using the sklearn.decomposition.NMF function. This resulted in a component matrix reflecting semantic topics and a loading matrix reflecting the loadings of each component onto each movie clip. The number of components was determined to obtain a simple model with each component being expressed in most of the movie clips (i.e., not too sparse in the loading matrix). We note that we accounted for a haemodynamic delay of 4 s when deriving the movie features. Each of the eight movie features and loadings of each semantic topic were then correlated to prediction performance scores across movie clips for each phenotype separately.

### 4.16 Additional Validation Analyses

We investigated the influence of subjects' head motion and eye movement measurements on phenotype prediction performance. Head motion was measured by mean FD across TRs for each subject. Three eye movement measurements were extracted from eye tracking data to reflect subject engagement levels, including blink rate, the number of fixations and the peak velocity of saccades. Blink rate was quantified as the number of blinks occurring during watching a given movie clip divided by its total number of TRs. The peak velocity was quantified as the median peak velocity over all saccades within each clip. For each measure, the values were averaged over subjects within each movie clip and then correlated with prediction performance scores across movie clips for each phenotype.

## Author Contributions

**Xuan Li** and **Susanne Weis:** designed the study. **Xuan Li:** performed research and data analysis. **Susanne Weis** and **Simon B. Eickhoff:** supervised the project and edited manuscript. **Xuan Li:** drafted the manuscript.

## Conflicts of Interest

Simon B. Eickhoff is the Editor‐in‐Chief of HBM and co‐author on this article, and he was excluded from the peer‐review process and all editorial decisions related to the publication of this article. Peer‐review was handled independently by a member of the HBM editorial board.

## Supporting information


**DATA S1** Supporting Information.

## Data Availability

The HCP data are publicly available and can be downloaded from the HCP website (https://db.humanconnectome.org/) and via Datalad (https://datasets.datalad.org/) (Halchenko et al. 2021). Family structure information can be accessed after approval of the HCP Restricted Data Use Terms. The code for phenotype prediction and movie feature extraction is available in the Github repository (https://github.com/xuanli‐ac/TOPF_movie_selection). Part of the code to perform TOPF was adapted from our previous code (https://github.com/xuanli‐ac/TOPF).
