# Supplementary material for: Stimulus Selection Influences Prediction of Individual Phenotypes in Naturalistic Conditions
Source: Hum Brain Mapp. 2025 Feb 17;46(3):e70164. doi: 10.1002/hbm.70164 (PMC11831449; doi:10.1002/hbm.70164)
Supplement: Supplementary file 1 — DATA S1 Supporting Information. [file HBM-46-e70164-s001.docx]

Supplementary Materials for

**Stimulus selection influences prediction of individual phenotypes in naturalistic conditions**

Xuan Li, Simon B. Eickhoff, Susanne Weis


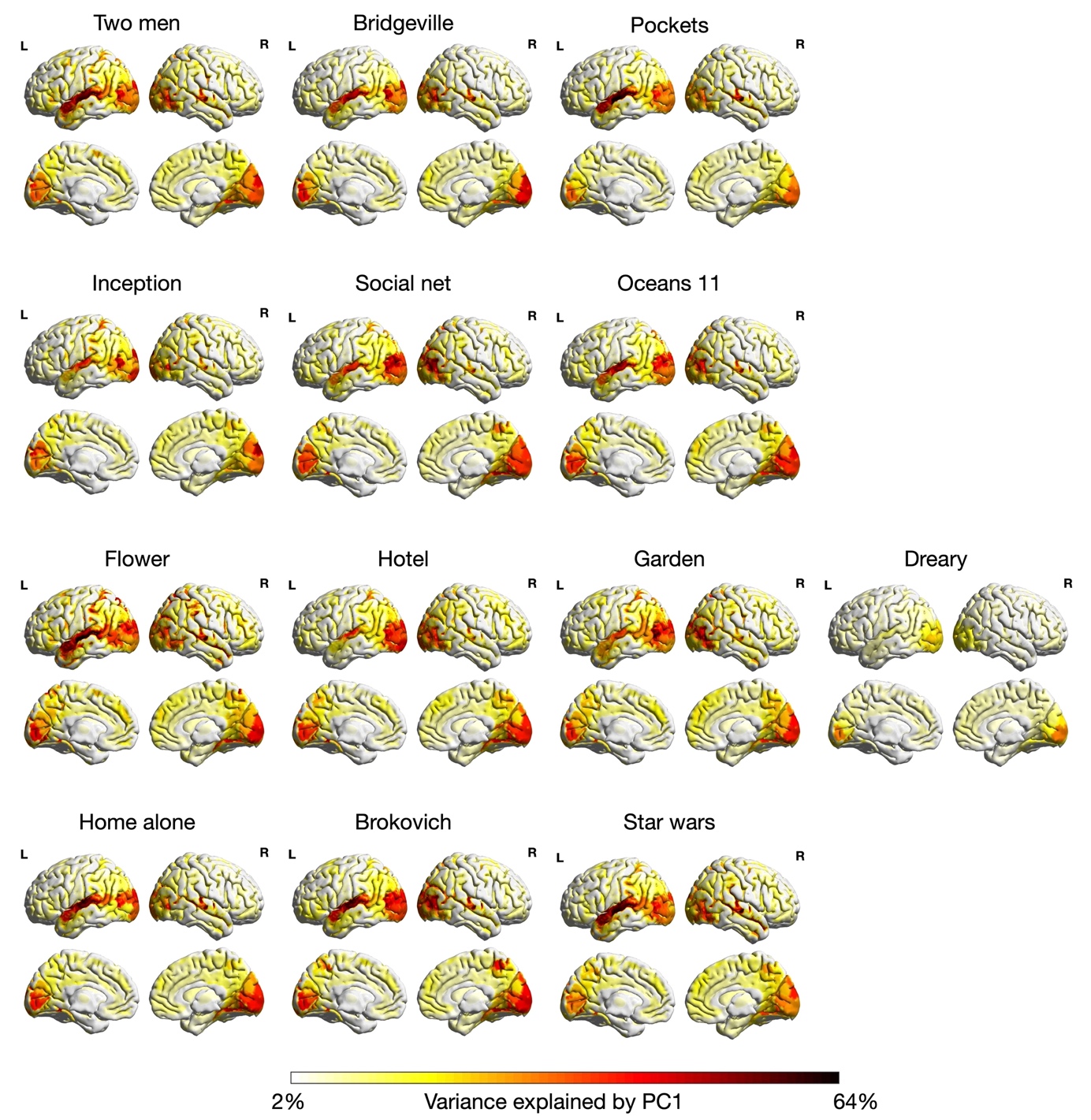


**Fig. S1: Brain maps of inter-subject synchrony for individual movie clips.** Each value represents the inter-subject synchrony of brain activity (quantified as the variance explained by PC1) in a given ROI. High and low inter-subject synchrony values are indicated by dark and light colours, respectively.


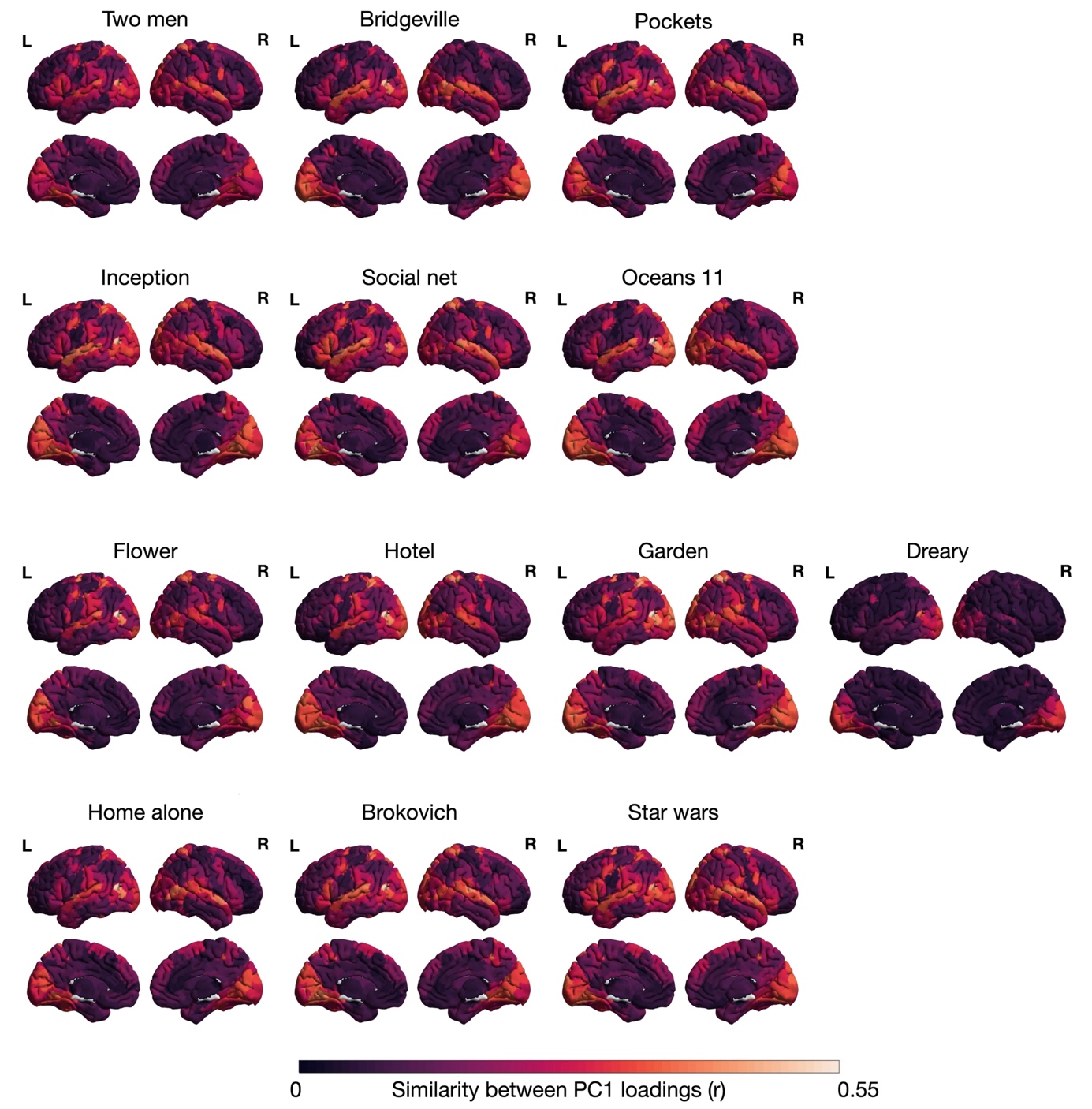


**Fig. S2: Between-movie similarity of inter-subject variability for individual movie clips.** Each value represents the average similarity (Pearson’s r) between one movie clip and all the other movie clips of PC1 loadings across subjects in a given ROI. Low and high between-movie similarity values are indicated by dark and light colours, respectively.


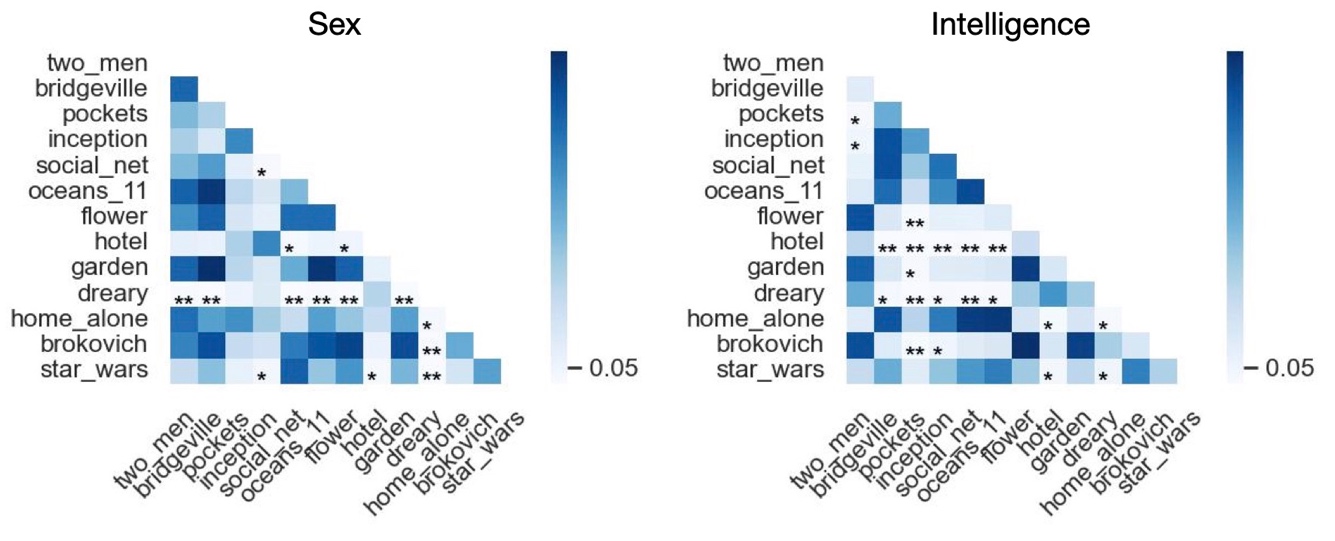


**Fig. S3: Comparison between movie clips by corrected resampled paired t-tests for sex classification accuracy and prediction performance of intelligence.** * denotes p<0.05. ** denotes p<0.01.

**
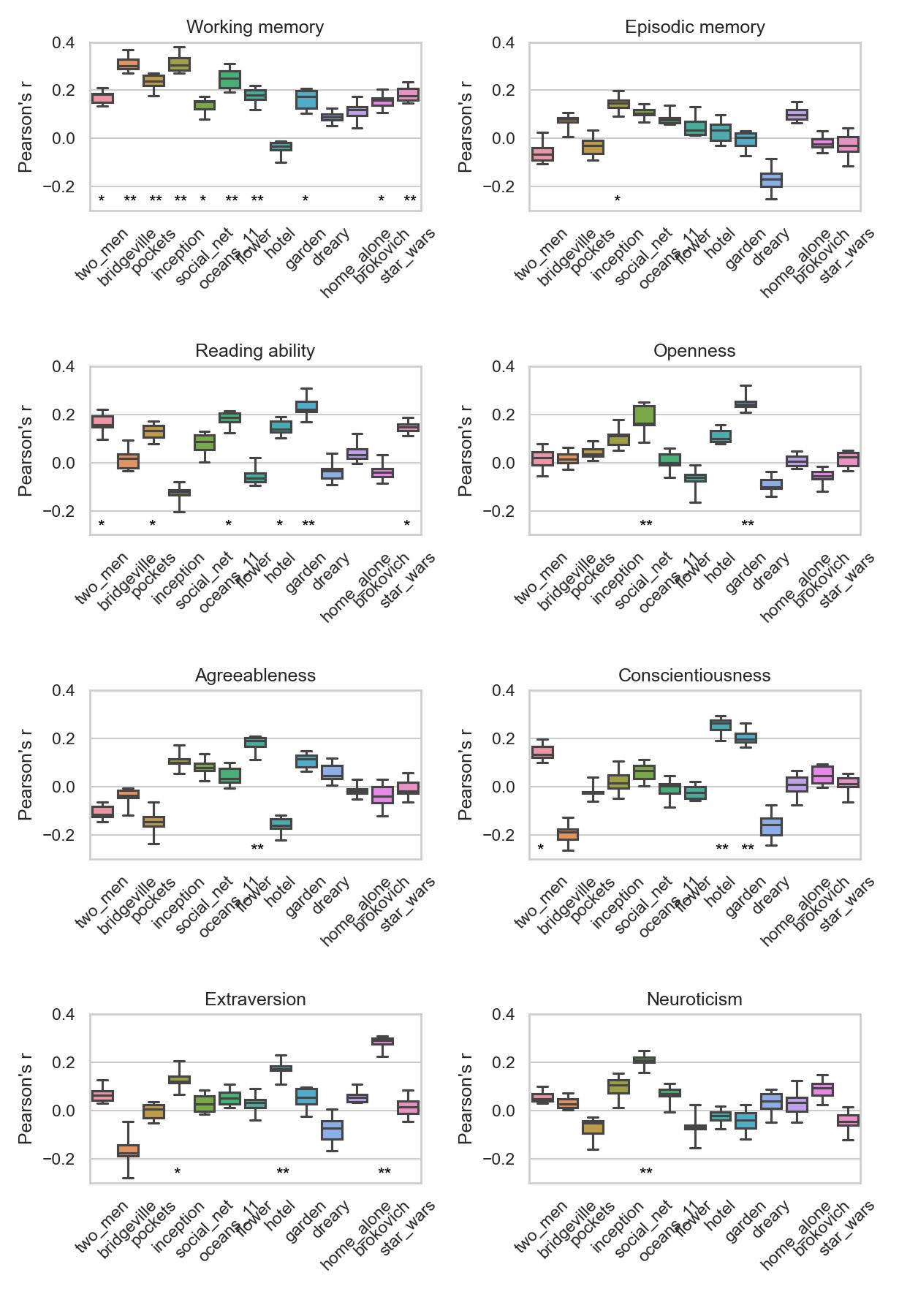
Fig. S4: Prediction performance for eight additional phenotypes.** The eight phenotypes reflect different aspects of cognitive abilities and personality. The boxplots show the scores (Pearson’s r between predicted and true phenotypic scores) from ten repetitions of cross validations. A permutation test (1000 iterations) was used to evaluate whether the performance score was significantly above chance. * denotes p<0.05 (FDR corrected). ** denotes p<0.01 (FDR-corrected).


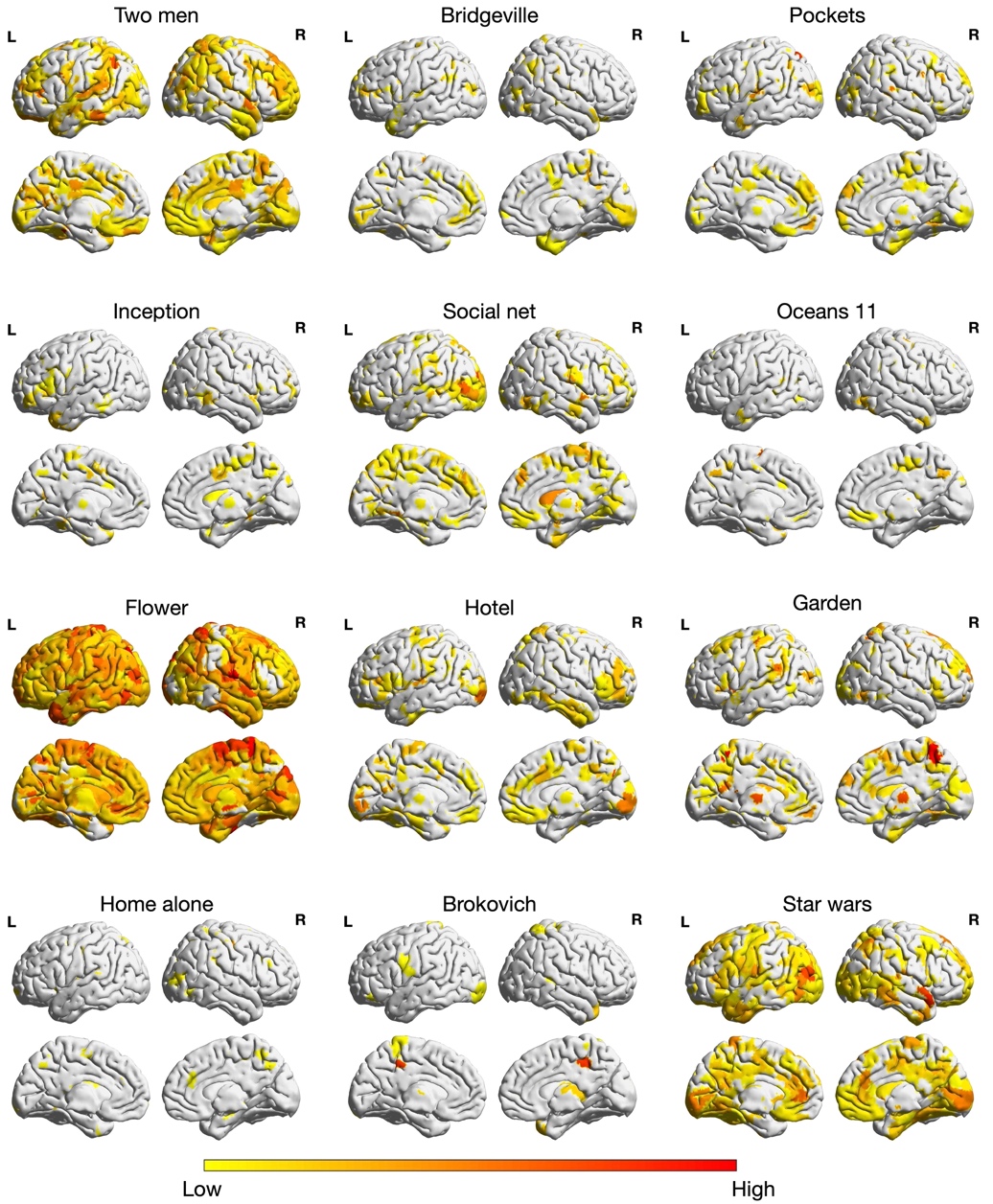


**Fig. S5: Feature importance for sex classification.** Permutation importance of each feature (brain region) was measured as the decrease in balanced accuracy after shuffling the feature across subjects. Each value in the brain maps represents the importance value averaged over 1000 permutation iterations and all models of a given clip. Only predictive features (with positive importance values) of movie clips having significant classification accuracies are shown. Low and high importance values are indicated by the colours yellow and red, respectively.


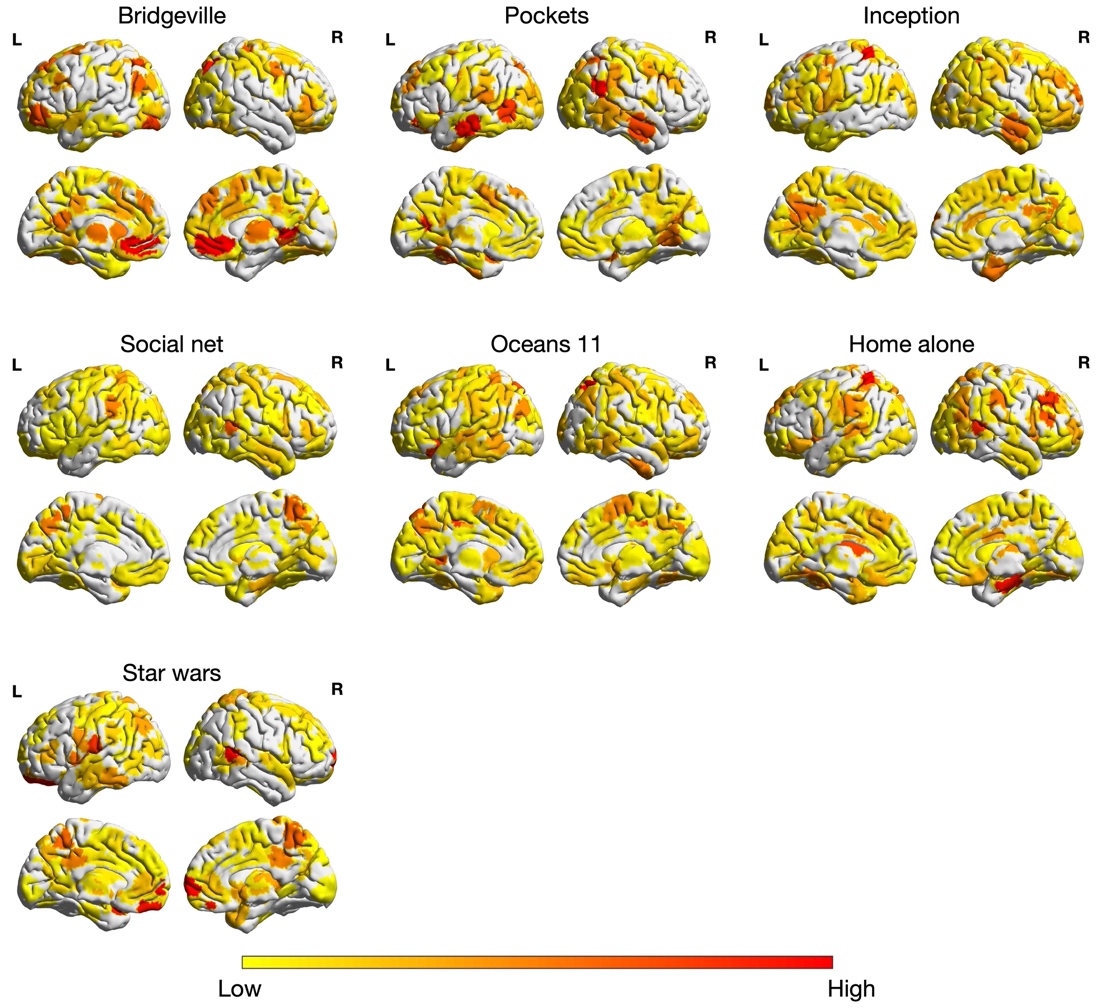


**Fig. S6: Feature importance for prediction of fluid intelligence.** Permutation importance was computed in the same way as for Fig. S3. Only predictive features (with positive importance values) of movie clips having significant predictions of fluid intelligence are shown. Low and high importance values are indicated by the colours yellow and red, respectively.


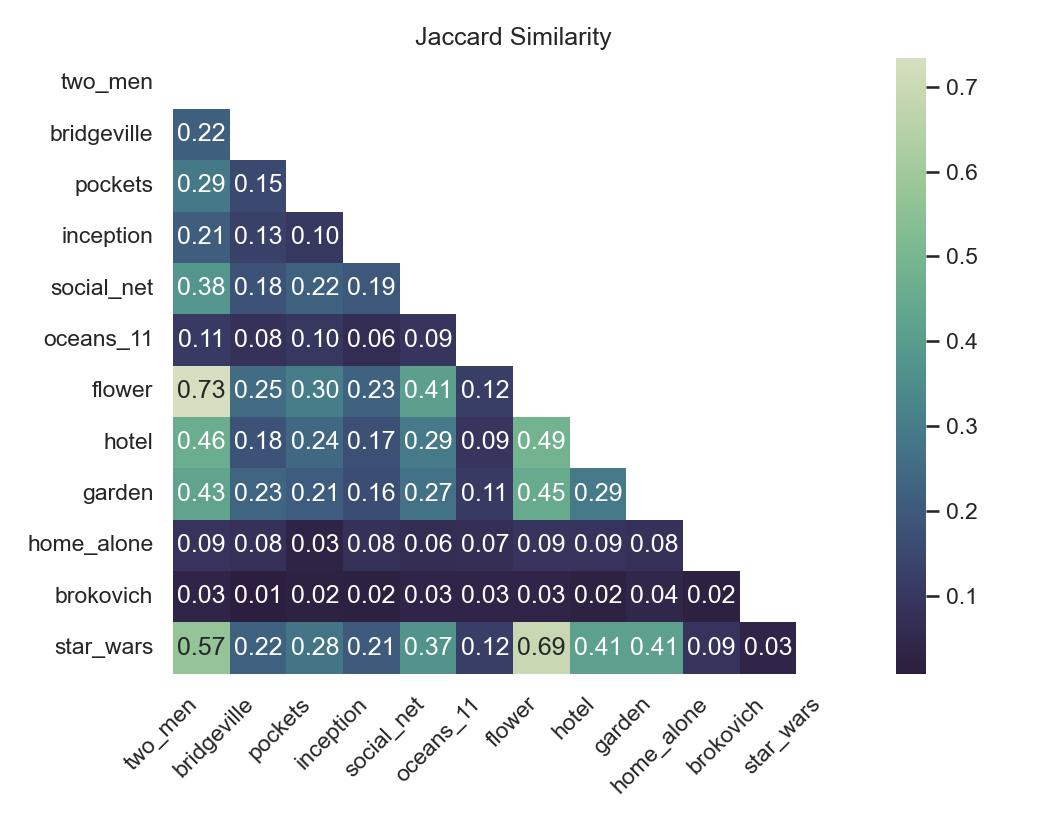


**Fig. S7: Similarity of predictive features for sex classification based on PC2 loadings.** For sex classification, both PC1 and PC2 loadings of all ROIs were used as features. Permutation feature importance was computed for each feature separately. Here, we show that the Jaccard similarity between movie clips of predictive features derived based on PC2 loadings was highly similar to that was derived based on PC1 loadings (Fig. 5B).

**
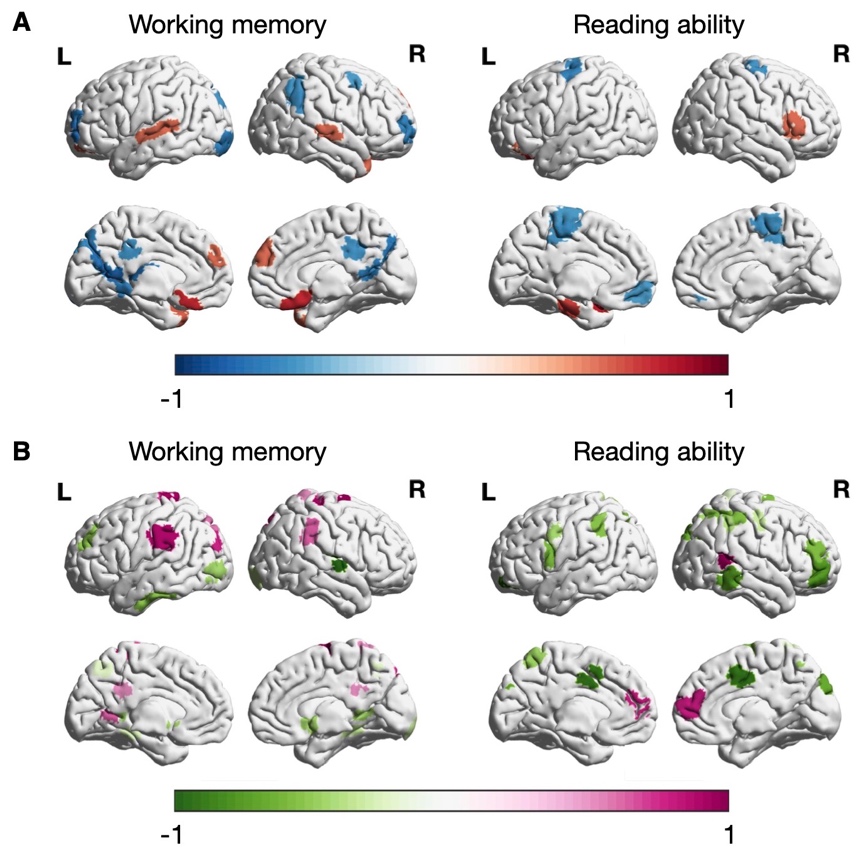
**

**Fig. S8: Influence of inter-subject synchrony on prediction performance of additional phenotypes.** **A**) Linear effect of synchrony on predictions. Brain regions exhibiting a linear effect of synchrony on prediction performance are shown here for each phenotype. Blue and red colours indicate negative and positive regression coefficients of the linear term, respectively. **B**) Quadratic effect of synchrony on predictions. Brain regions exhibiting a quadratic effect of synchrony on prediction performance are shown here for each phenotype. Green and violet colours indicate negative and positive regression coefficients of the second-order term, respectively. Note that, to ensure a meaningful analysis, only working memory and reading ability that were best predicted overall were included here (Fig. S4).

**
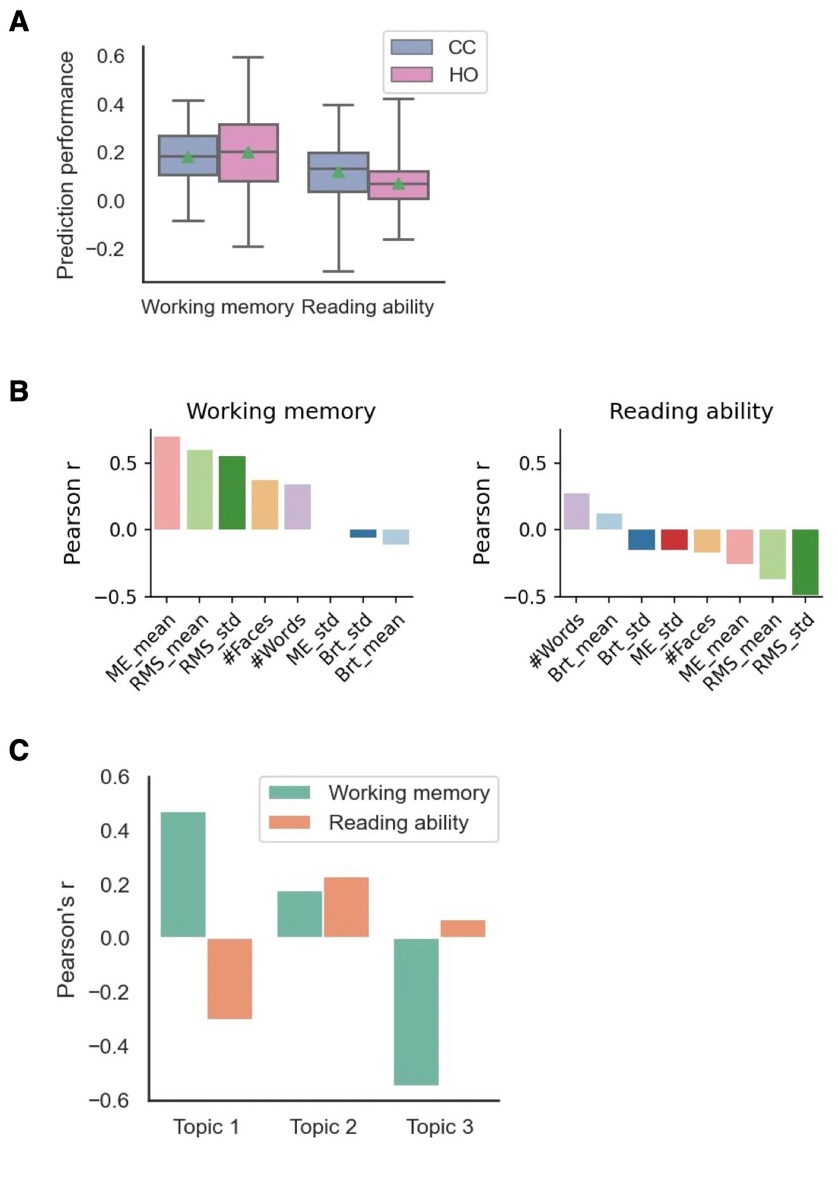
**

**Fig. S9: Influence of multi-level features of movie stimuli on prediction performance for working memory and reading ability.** **A**) Comparison between independent (CC) and Hollywood (HO) movie clips in prediction performance. The boxplot convention is consistent with that used in Fig. 7A (green triangles: means). No significant differences were found between CC and HO for both phenotypes. **B**) Correlation (Pearson’s r) between movie features and prediction performance across movie clips for working memory and reading ability separately. **C**) Correlations between prediction performance and loadings of each semantic topic across movie clips for each phenotype.


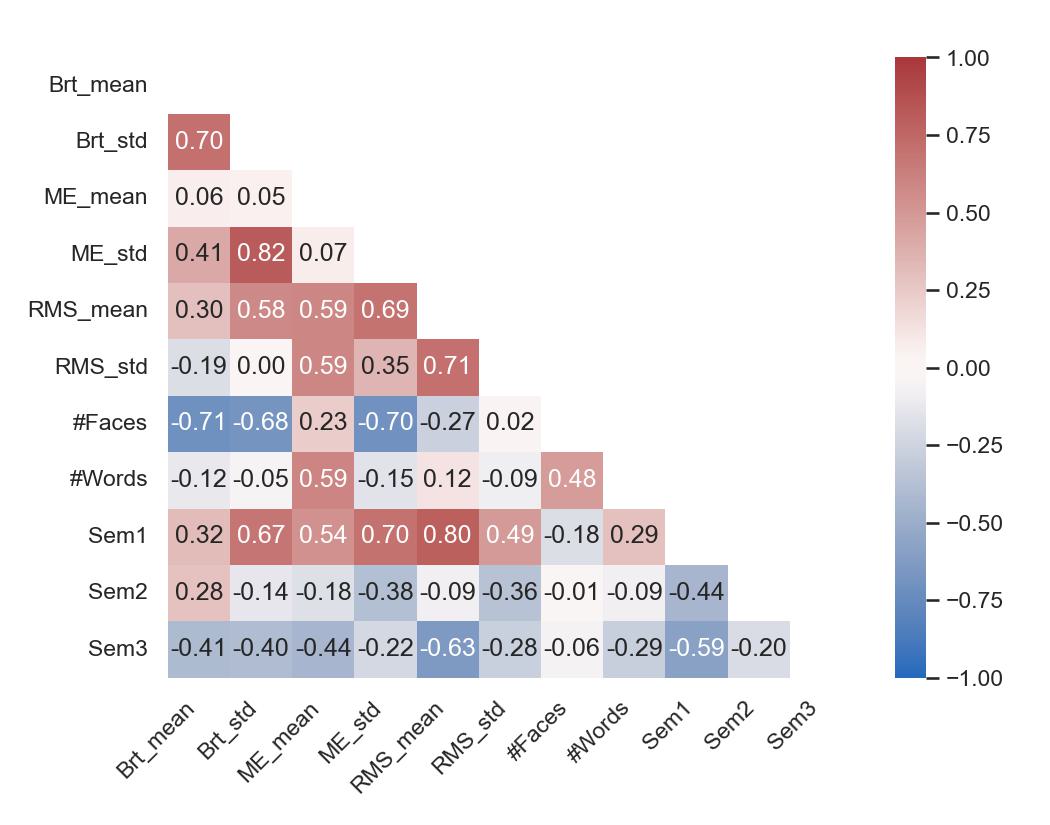


**Fig. S10: Correlation coefficients between different movie features.** For each pair of movie features, the (Pearson’s) correlation coefficient was computed over the values of all movie clips (excluding “dreary”). In addition to the eight low- and middle-level features, we also included three high-level semantic features, i.e., Sem1, Sem2 and Sem3. They denote the three semantic topics (Fig. 7C) derived from the semantic labels separately.


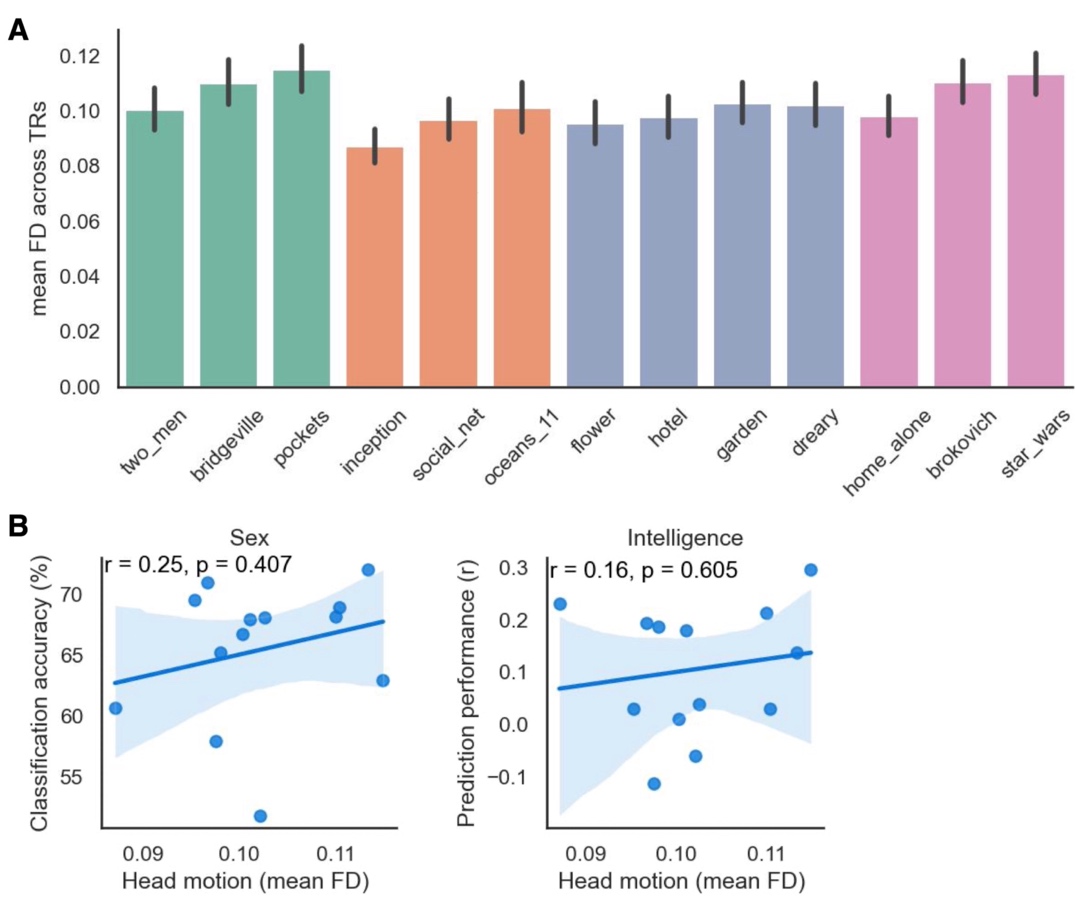


**Fig. S11:** **Influence of head motion on prediction performance.** **A**) Head motion (mean FD across TRs) for each movie clip. The bars indicate the average head motion over all subjects, with each error bar reflecting the standard deviation across subjects. The movie clips are shown in the same order as they were presented within each run from run 1 to run 4. Movie clips belonging to the same run are marked in the same colour. **B**) Scatter plot of the relationship (Pearson’s r) between head motion and prediction performance over movie clips, for sex and fluid intelligence separately. Each dot represents a movie clip.


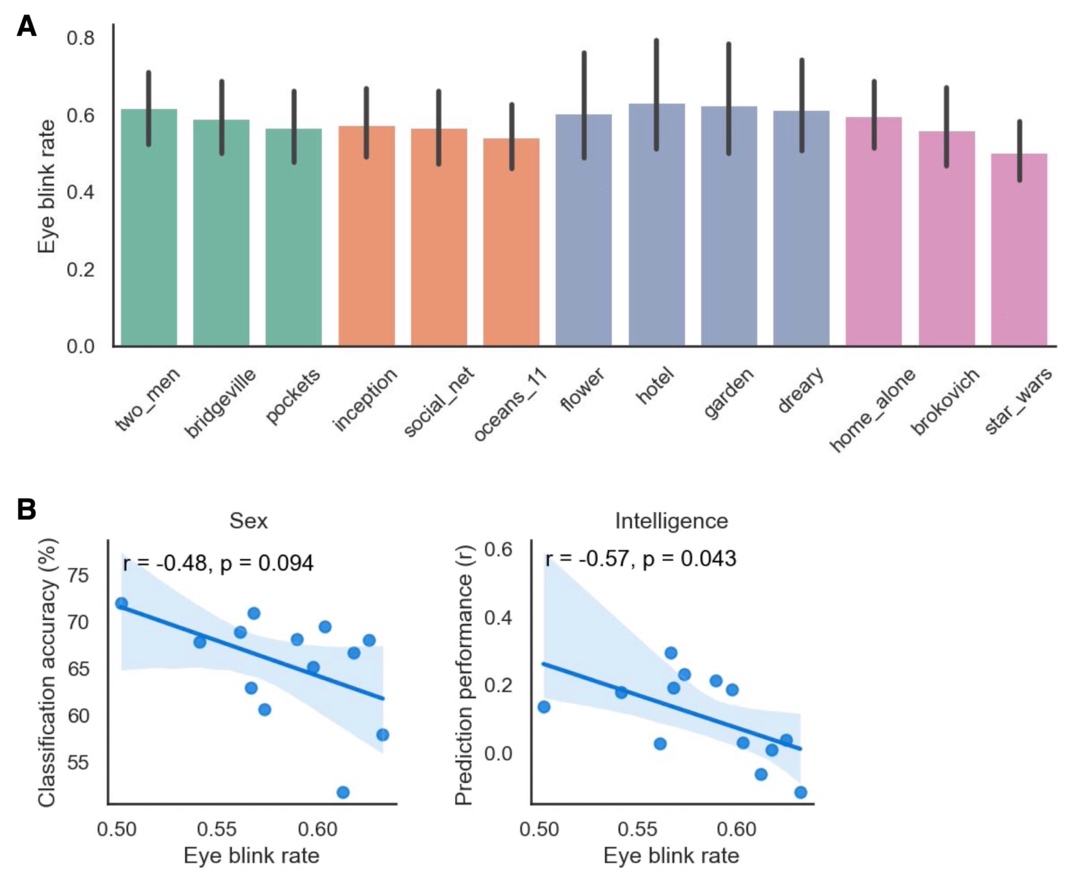


**Fig. S12:** **Influence of eye blink rate on prediction performance.** **A**) Eye blink rate for each movie clip. The bars indicate the average over all subjects, with each error bar reflecting the standard deviation across subjects. The movie clips are shown in the same order as they were presented within each run from run 1 to run 4. Movie clips belonging to the same run are marked in the same colour. **B**) Scatter plot of the relationship (Pearson’s r) between eye blink rate and prediction performance over movie clips, for sex and fluid intelligence separately. Each dot represents a movie clip.


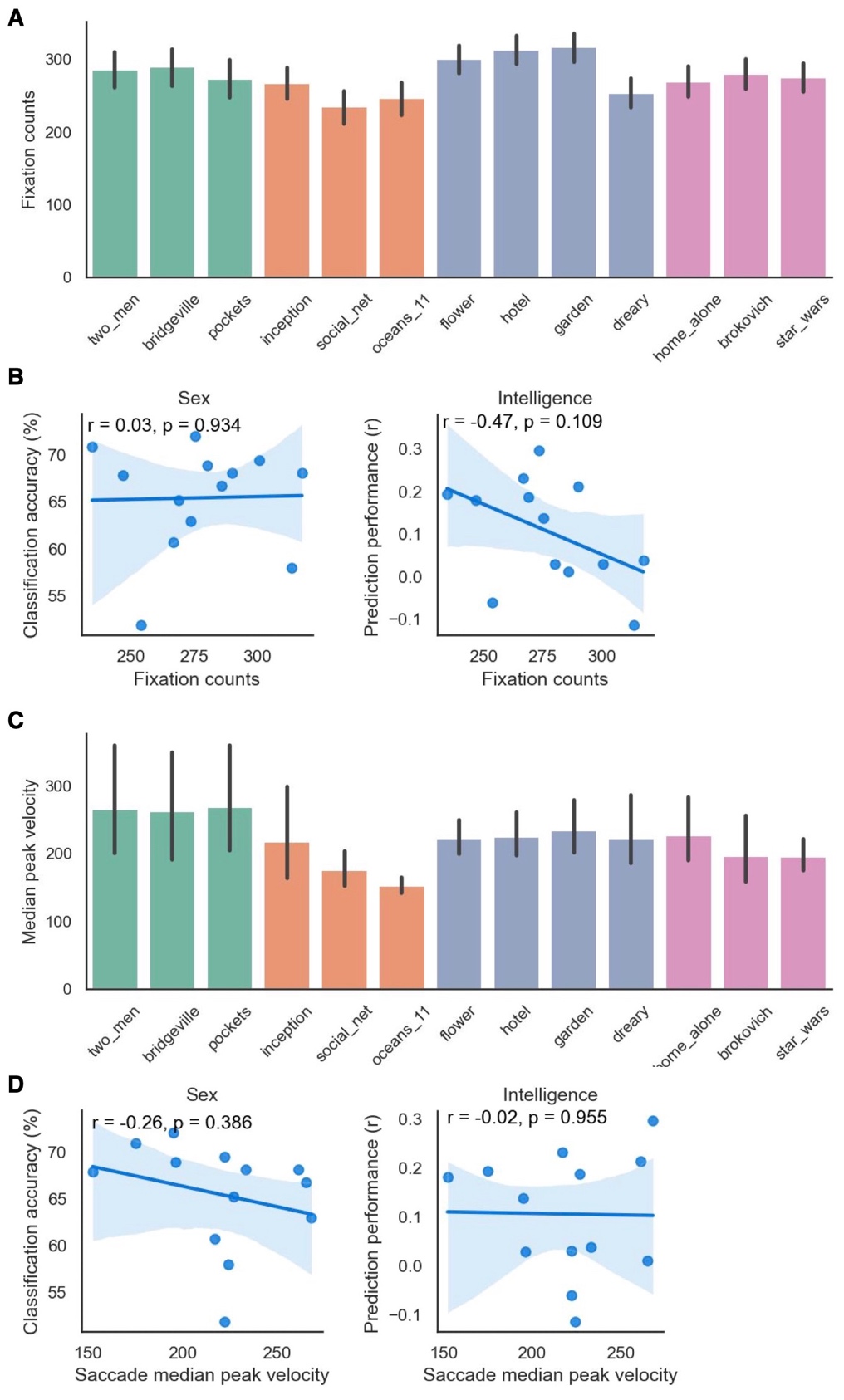


**Fig. S13: Influence of additional eye movement measurements on prediction performance.** **A**) The number of fixations for each movie clip. The bars indicate the average over all subjects, with each error bar reflecting the standard deviation across subjects. The movie clips are shown in the same order as they were presented within each run from run 1 to run 4. Movie clips belonging to the same run are marked in the same colour. **B**) Scatter plot of the relationship (Pearson’s r) between the number of fixations and prediction performance over movie clips, for sex and fluid intelligence separately. Each dot represents a movie clip. **C**) The median peak velocity of saccades for each movie clip. **D**) Scatter plot of the relationship (Pearson’s r) between the median peak velocity and prediction performance over movie clips, for sex and fluid intelligence separately. Plot conventions for C) and D) are consistent with those for A) and B), respectively.

| Run | type | Short name | Original name | Abbr. | TRs | Duration  (min:sec) | Content |
| --- | --- | --- | --- | --- | --- | --- | --- |
| **1** | CC | Two Men | Two Men | tm | 234 | 3:54 | A man is observing another man running in his direction, while narrating his thoughts and thinking whether he should intervene. The narrator speaks accented English and subtitles are given. The last 32 TRs show the credits accompanied by music. |
| **1** | CC | Bridgeville | Welcome to Bridgeville | b | 211 | 3:31 | Inhabitants of a town tell stories about them enjoying living there. The speakers are shown with montages of the town and accompanied by music. |
| **1** | CC | Pockets | Pockets | p | 179 | 2:59 | People show what they keep in their pockets and explain their personal meaning. Clips show close-ups of their items and faces, accompanied by music. The last 9 TRs show credits. |
| **2** | HO | Inception | Inception | i | 217 | 3:37 | A man and a woman walk around at an imaginary location, learning how to interact with their surroundings and its possible dangers. |
| **2** | HO | Social Net | The Social Network | sn | 249 | 4:09 | The clip consists of many short scenes. It shows young Zuckerberg at university at a hearing where he is being accused of breaking rules. The last scene shows Zuckerberg leaving a lecture emotionally agitated. The scenes are re-enacted. |
| **2** | HO | Ocean’s 11 | Ocean’s Eleven | o | 239 | 3:59 | A man introduces his later accomplices to his plan on how to rob a casino. The men discuss their approach. |
| **3** | CC | Flower | Off the Shelf | f | 170 | 2:50 | A song with montage of a flower and its journey through different places |
| **3** | CC | Hotel | 1212 | h | 174 | 2:54 | A man and a woman are in a hotel room and supernaturally experience the presence of each other but never meet. |
| **3** | CC | Garden | Mrs Meyer’s Clean Day | g | 195 | 3:15 | A documentary of a woman presenting her community program teaching people how to grow food. |
| **3** | CC | Dreary | Northwest Passage | d | 132 | 2:12 | ﻿Montage of landscapes and objects (e.g., houses and trucks) in a foggy and dusty atmosphere, with freaky background music. |
| **4** | HO | Home Alone | Home Alone | ha | 222 | 3:42 | A boy walks through his parents’ house looking for his family, realising that he is home alone. |
| **4** | HO | Brockovich | Erin Brockovich | bv | 220 | 3:40 | A woman visits another woman asking her to testify at court. Afterwards she visits her place of work, a legal office, accompanied by her children. |
| **4** | HO | Star Wars | The Empire Strikes Back | sw | 246 | 4:06 | A man riding through an icy landscape gets attacked by a beast. People and imaginary animals work on aircrafts. A man and a woman are fighting. |

**Table S1:** **Summary of the 13 movie clips in HCP used in our analyses.** CC and HO denote independent films and Hollywood films, respectively. Abbr. denotes the abbreviation of the name of each clip. Note that in the given durations the first 10 TRs were already excluded for each movie clip.

| **Movie** | **Brt_mean** | **Brt_std** | **ME_mean** | **ME_std** | **RMS_mean** | **RMS_std** | **#FaceTRs** | **#Words** |
| --- | --- | --- | --- | --- | --- | --- | --- | --- |
| Two Men | 0.3586 | 0.1011 | -0.1151 | 0.3587 | 0.0212 | 0.0180 | 46 | 133 |
| Bridgeville | 0.4561 | 0.1162 | 0.1676 | 0.2764 | 0.0223 | 0.0132 | 111 | 283 |
| Pockets | 0.5373 | 0.0795 | -0.0737 | 0.1899 | 0.0161 | 0.0053 | 69 | 137 |
| Inception | 0.2280 | 0.0414 | 0.3430 | 0.2235 | 0.0293 | 0.0315 | 155 | 170 |
| Social Net | 0.1408 | 0.0394 | -0.1046 | 0.1991 | 0.0069 | 0.0056 | 192 | 310 |
| Ocean's 11 | 0.1932 | 0.0470 | 0.0444 | 0.1546 | 0.0097 | 0.0102 | 154 | 280 |
| Flower | 0.4892 | 0.2109 | -0.0150 | 0.4367 | 0.0335 | 0.0153 | 2 | 121 |
| Hotel | 0.4529 | 0.0869 | -0.3313 | 0.2082 | 0.0026 | 0.0045 | 49 | 42 |
| Garden | 0.5065 | 0.1181 | 0.3445 | 0.2927 | 0.0225 | 0.0118 | 59 | 469 |
| Dreary | 0.3873 | 0.1573 | -0.1603 | 0.3450 | 0.0066 | 0.0000 | 0 | 0 |
| Home Alone | 0.2418 | 0.0506 | -0.0896 | 0.2485 | 0.0062 | 0.0118 | 52 | 74 |
| Brockovich | 0.4968 | 0.1106 | 0.1127 | 0.2273 | 0.0103 | 0.0088 | 97 | 186 |
| Star Wars | 0.5038 | 0.1007 | 0.0765 | 0.3168 | 0.0204 | 0.0168 | 17 | 79 |

**Table S2:** **Eight low- and middle-level features of the 13 movie clips.** For brightness (Brt), motion energy (ME), and audio loudness (root-mean-square, RMS), the mean and standard deviation across TRs were computed. #FaceTRs and #Words denote the total number of TRs showing human faces and the total number of words spoken within each movie clip, respectively.
